# Diverging neural dynamics for syntactic structure building in naturalistic speaking and listening

**DOI:** 10.1073/pnas.2310766121

**Published:** 2024-03-05

**Authors:** Laura Giglio, Markus Ostarek, Daniel Sharoh, Peter Hagoort

**Affiliations:** ^a^Max Planck Institute for Psycholinguistics, Nijmegen 6525XD, The Netherlands; ^b^Radboud University, Donders Institute for Brain, Cognition and Behaviour, Nijmegen 6525EN, The Netherlands

**Keywords:** language production, language comprehension, syntax, fMRI, naturalistic

## Abstract

The neural correlates of sentence production are typically studied using task paradigms that differ considerably from the experience of speaking outside of an experimental setting. In this fMRI study, we aimed to gain a better understanding of syntactic processing in spontaneous production versus naturalistic comprehension in three regions of interest (BA44, BA45, and left posterior middle temporal gyrus). A group of participants (n = 16) was asked to speak about the events of an episode of a TV series in the scanner. Another group of participants (n = 36) listened to the spoken recall of a participant from the first group. To model syntactic processing, we extracted word-by-word metrics of phrase-structure building with a top–down and a bottom–up parser that make different hypotheses about the timing of structure building. While the top–down parser anticipates syntactic structure, sometimes before it is obvious to the listener, the bottom–up parser builds syntactic structure in an integratory way after all of the evidence has been presented. In comprehension, neural activity was found to be better modeled by the bottom–up parser, while in production, it was better modeled by the top–down parser. We additionally modeled structure building in production with two strategies that were developed here to make different predictions about the incrementality of structure building during speaking. We found evidence for highly incremental and anticipatory structure building in production, which was confirmed by a converging analysis of the pausing patterns in speech. Overall, this study shows the feasibility of studying the neural dynamics of spontaneous language production.

## Significance

Neuroimaging studies of language processing usually focus on language comprehension. This is because language production is affected by increased motion artifacts and is challenging to control experimentally. Sentence production studies typically rely on task designs that impose strong constraints on speaking. Here, we studied the brain responses to syntactic structure building during spontaneous production and naturalistic comprehension. We found brain responses to be sensitive to structure building in both production and comprehension, but with different temporal profiles in each modality. In production, the structure was built early in a sentence in an anticipatory way, while in comprehension structure building followed the input and was thus integratory. These results highlight different dynamics of syntactic structure building during speaking and listening.

Studies on the neurobiology of language typically use highly controlled experimental paradigms that are far removed from the everyday experience of language use. The last decade, however, has seen a relative increase in the number of studies investigating naturalistic language processing. These studies are diverse in their methodologies, from the use of virtual environments (1, 2), to the auditory presentation of audiobooks or narrative reading with neuroimaging (3–7). The increased ecological validity in naturalistic studies opens a window into language processing free of the artificiality of task designs, whose main goal is to isolate specific features of language (8). In traditional settings, experimental control comes at the cost of context, which is reduced to minimize confounds. This contrasts with the highly contextual nature of everyday language use, creating a large gap between the actual object of study and its realization in experiments. Combining naturalistic stimuli and advanced analysis methods, such as audiobooks and probabilistic parsers, has the potential to bring the participant experience during a language experiment closer to the experience of everyday language use (4, 9).

In addition to the predominant use of context-reduced experiments, the majority of studies on the neurobiology of language focuses on comprehension, while speaking is relatively unexplored (e.g., meta-analyses on sentence production and comprehension have at least three times as many studies of comprehension (10, 11)). Importantly, while naturalistic studies are becoming more common in the field of language comprehension, studies of naturalistic production are still rare.^*^ This is problematic because of the large gulf between spontaneous production and production in controlled experiments. In spontaneous language production, the speaker is by definition in control of what is said. In contrast, experimental paradigms attempt to have as much control over participants’ speech as possible. This has usually been achieved with picture description experiments or with the use of visual probes together with written linguistic stimuli (12–15). While these strategies have allowed for controlled investigations of linguistic processing, they may be confounded by task requirements that make controlled production very different from everyday speaking.

In this functional magnetic resonance imaging (fMRI) study, we aimed to study syntactic processing in spontaneous production and comprehension in order to understand whether and how they differ. Peripherally, production and comprehension are obviously different as they arguably constitute the opposite ends of linguistic processing. Accordingly, they are grounded in two systems, the articulatory-motor system and the auditory system, which are clearly separate. Aside from these differences in peripheral processes, the linguistic items that comprehension and production interface with are the same. However, traditionally production and comprehension have been thought to be grounded in separate processing and neural systems (e.g., refs. 16 and 17), due to different developmental trajectories (18) and linguistic impairments following stroke (19, but see refs. 20 and 21). More recent studies have found shared neural resources for production and comprehension (22, 23) and a similar network for processing syntactic complexity across modalities (12, 24), which supports the view that syntactic representations are shared across modalities, since syntactic priming effects are resistant to modality changes (25) and lead to neural adaptation across modalities (23).

Syntactic processing in comprehension has been studied in the last decade with word-by-word indices of processing load that build syntactic trees from hypothesized syntactic operations (4, 9). Increasingly sophisticated approaches that characterize incremental structure building during listening and reading have made clear that left fronto-temporal regions are sensitive to measures of syntactic tree building (26–31). Many of these approaches quantified syntactic structure building using a top–down and a bottom–up parser strategy (27, 30, 32). These strategies account for the same structure but make different hypotheses about the timing of syntactic operations. The top–down parser builds nodes at phrase-opening in an anticipatory fashion, sometimes anticipating the structure before it is unambiguous to the listener. The bottom–up parser instead builds nodes at phrase-closing in an integratory fashion, when the structure can be built unambiguously. Here, we asked whether these incremental measures of structure building would also be able to track neural activity during spontaneous production. Given the existing evidence for shared syntactic representations between production and comprehension, we hypothesized that these strategies would be suitable for production, since similar structures are expected to be built in production and comprehension.

It is instead less clear whether the processes that build syntactic representations are shared between production and comprehension. Behavioral evidence in favor of shared processes shows that syntactic structure building in production interferes with parsing in comprehension, which is argued to be possible only if they rely on a common processor (33). It is therefore reasonable to assume that similar processes underlie the building of syntactic structure in production and comprehension. These shared processes, though, may unfold with different temporal dynamics. The context, or amount of knowledge available to the syntactic encoder, may differ between modalities. In production, the speaker has some knowledge about the upcoming structure, since the structure related to the words that are uttered must have been computed (34, 35). In comprehension, instead, after accounting for predictable continuations, listeners need to wait for the input to fully or correctly compute the structure, as shown for example by garden path sentences (36–38). This processing difference may have consequences for the way these parsers model neural activity in production and comprehension, since effectively they make different hypotheses about the timing of structure building. Therefore, we hypothesized that the timing of syntactic operations would be the critical difference between production and comprehension, due to the different requirements and inputs of each modality (39). We thus expected neural activity to increase in production in relation to anticipatory top–down operations, due to the speaker’s planning of upcoming structure. Instead, we expected that bottom–up operations would predict an increase in neural activity in comprehension, where listeners need to wait for the input to commit to a structure. In a follow-up exploratory analysis, we assessed whether alternative parsing strategies may be more fitting for production, since the parser models discussed so far were mainly discussed in the context of comprehension and were relatively less prevalent in the generation literature (40). In particular, we developed two parsers that assume different levels of incremental processing, by making different predictions about how early phrase-structure building operations occur.

Finally, we investigated responses to syntactic processing in three regions of interest (ROIs): BA44, BA45 (*pars opercularis* and *pars triangularis* of the left inferior frontal gyrus (LIFG)), and the left posterior middle temporal gyrus (LpMTG). We focused on these three regions because of their previously observed involvement in syntactic processing and their critical role for syntactic processing according to several models (41–44). These regions were all found to respond to syntactic manipulations in both modalities in previous studies (15, 32, 44–48), sometimes with differences in their sensitivity to each modality (11, 12, 49). In particular, the LIFG was found to be more responsive to syntactic manipulations in production than comprehension (12, 50), while the LpMTG was more responsive during comprehension (12, 49). Although other regions may have been responsive to these predictors, as suggested by previous results (e.g., refs. 27 and 30), we preferred to only include ROIs that have been most consistently associated with syntactic processing, to preserve interpretability and statistical power. This approach made it possible to include ROI as a fixed effect, which allowed us to assess differences between ROIs statistically. To summarize, we investigated whether word-by-word indices of syntactic processing that were previously seen to track neural activity in comprehension would be suitable for production in three ROIs previously associated with syntactic processing.

## Results

### Incremental Metrics of Phrase-Structure Building.

**Fig. 1.**
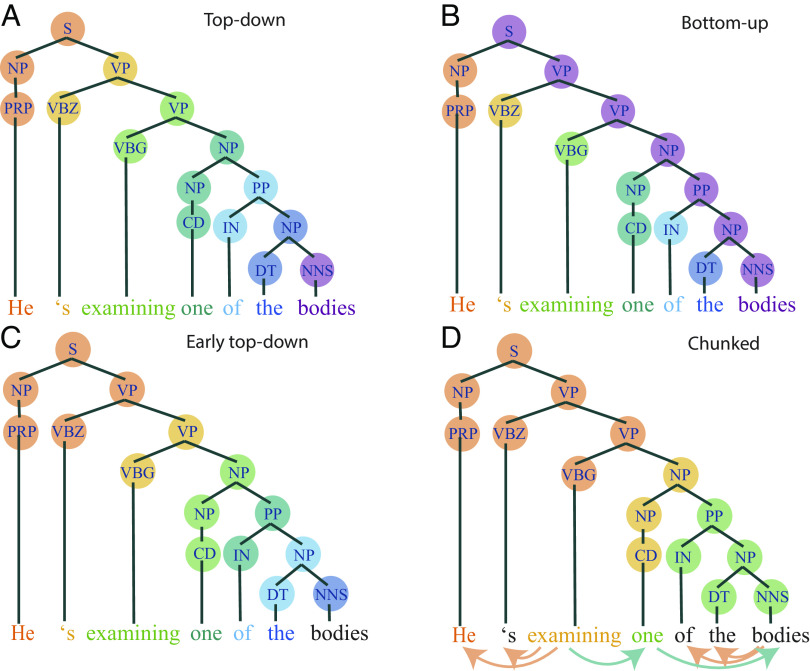
Node counting following different parsing strategies. The colored circles refer to the nodes that are built at the time point the word in the same color is uttered or heard. (*A*) Colored representation of top–down phrase structure building, with nodes counted from the top of the tree to the word. (*B*) Colored representation of bottom–up phrase structure building, with nodes counted from the bottom of the tree (i.e. the terminal nodes) to the top. Only nodes where both daughter nodes have been already met can be counted at each word. (*C*) Colored representation of early top–down phrase-structure building, assuming operations to take place before word onset (production-specific). (*D*) Colored representation of chunked phrase-structure building, following a less incremental strategy (production-specific). This node counting strategy is chunked based on the heads of the dependency parse of the same sentence (shown by the arrows below words, also see *SI Appendix*, Fig. S1). Heads are words from which an arrow originates. The nodes of the same constituent structure used by the other strategies are counted here, but they are assigned only to the first word and to subsequent heads. The chunked nature of this parser results in phrase-structure building operations assigned to some but not all words in a sentence (*SI Appendix*). Black words are words that are not assigned any phrase-structure building operation (e.g., sentence-final words).

To obtain incremental metrics of syntactic processing, we proceeded in two steps. First, we extracted the constituent structure of each sentence with a probabilistic context-free phrase-structure grammar (Stanford parser (51)). From the extracted constituent parse, we then computed the parser operations carried out at each word according to different parsing models (52). These parsers incrementally build the syntactic structure of a sentence following different strategies, leading to a hypothesized number of phrase-structure building operations that need to be carried out at each word (52). This results in an incremental complexity metric that corresponds to the number of nodes that are built with each word. A top–down strategy builds the phrase structure from the top of the tree to a given word, such that it predicts increased activity when phrases are opened. For comprehension, the top–down parser sometimes anticipates nodes before they are unambiguous to the listener, for example in the presence of adjuncts. Bottom–up parsing instead builds the phrase structure only after all the evidence has been heard, that is, after all words attached to each node have been met. It thus predicts increased activity when phrases are closed. Ultimately, both strategies lead to the same node count, but they make different predictions about the timing of syntactic operations and thus of corresponding neural activation (see *Methods* for more details, Fig. 1 *A* and *B*). It should be noted that the number of parser operations carried out at each word serves as an index of syntactic processing load throughout the sentence, rather than as a hypothesis about the actual computations taking place in the ROI.

We also quantified the load of processing complexity on working memory with an *open nodes* measure. This measure counts the number of nodes that have been opened (i.e., counted by the top–down strategy) but have not been closed yet (i.e., counted by the bottom–up strategy), tracking the numbers of words that need to be kept in working memory until they can be merged in a constituent (30). In other words, this complexity metric tracks how much of the hypothesized structure needs to be confirmed by upcoming input. We expected this index to predict an increase in activity in comprehension, following Nelson et al. and Uddén et al. (30, 53). In production, it would also lead to an activity increase if speakers kept track of the structure that remained to be closed. Finally, to make sure that the syntactic predictors did not simply track word probabilities based on context, we quantified word surprisal from transformer model GPT-2 (54).

### Distinct Dynamics for Phrase-Structure Building in Language Production vs. Comprehension.

We compared word-by-word predictors of syntactic structure building in spontaneous production and comprehension using two datasets shared on OpenNeuro (55, 56). In the first dataset, participants (n = 16) recalled the events of a TV series they had just watched in the scanner. This was the production condition. In the second dataset, participants (n = 36) listened to the recall of one production participant from the first dataset. The linguistic stimuli were thus very similar between the production and comprehension datasets, but modality was a between-subject variable.

**Fig. 2.**
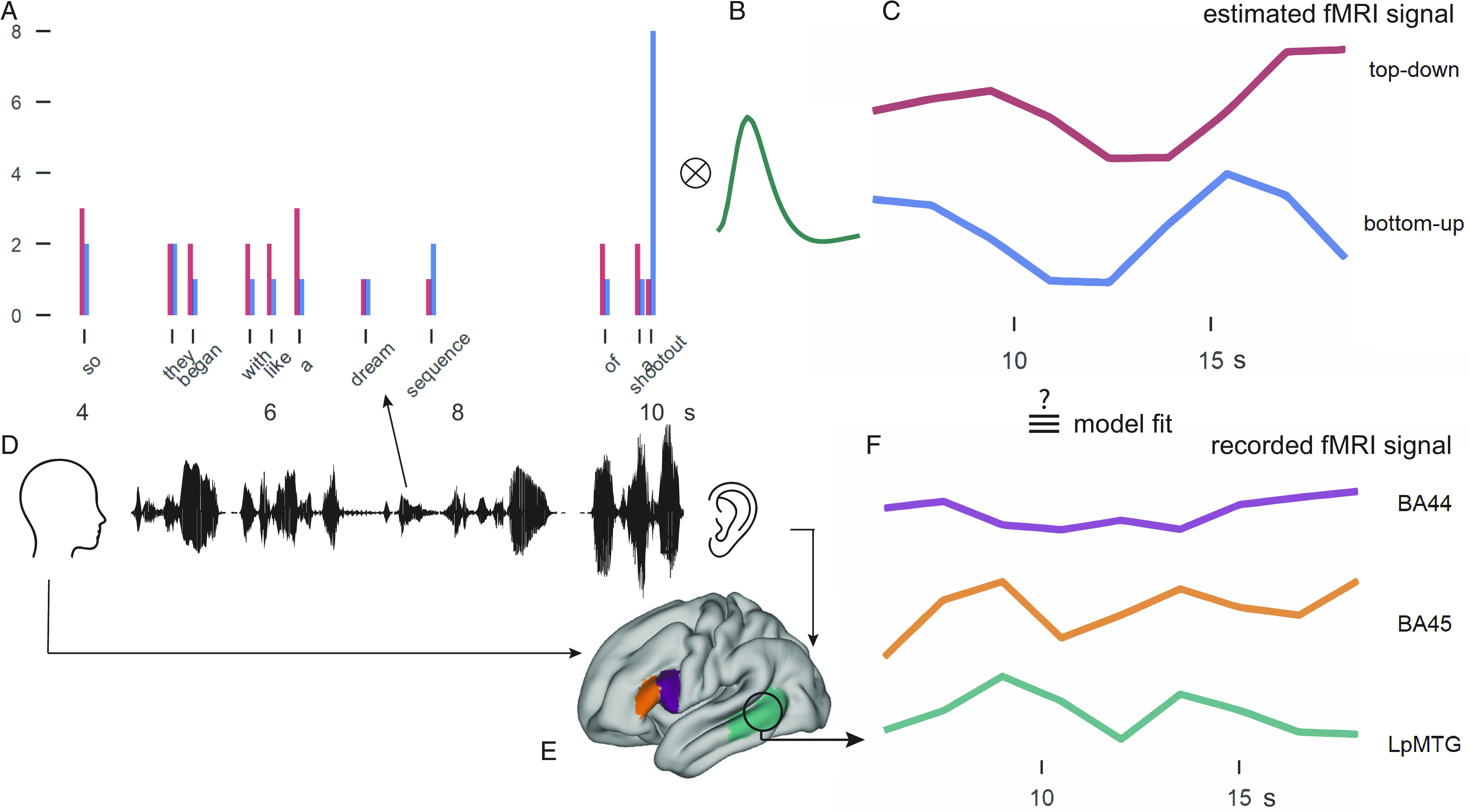
Graphical representation of the analysis procedure to relate word-by-word predictors of linguistic complexity to BOLD activity. (*A*) Word-by-word predictors of syntactic complexity were extracted from the constituent structure of the sentence spoken by a participant and listened to by other participants. The height of the bars in (*A*) represents the number of phrase-structure building operations expected to take place at each word following top–down and bottom–up parsing strategies (e.g., at “so” 3 nodes are counted for top–down, 2 for bottom–up). The weights of the syntactic predictors were convolved with the hemodynamic response function (*B*) to get predictor timeseries of BOLD activity at 1.5 s resolution (*C*). These predictors timeseries were then compared to the brain activity of the speaker or the listener (*D*) in the three ROIs (BA44, BA45, and LpMTG, *E*) extracted as average BOLD time courses (*F*).

To directly compare the word-by-word predictors with BOLD activity with a 1.5-s resolution (thus including several words at each fMRI volume), we convolved the linguistic predictors with the hemodynamic response function and resampled it to the 1.5 s repetition time (see *Methods* for more details, Fig. 2). We then regressed the average BOLD activity in BA44, BA45, and LpMTG in subject space against the predicted timeseries for each linguistic predictor with a linear mixed-effects model. The model included word rate, syllable rate, word frequency, word surprisal, open nodes, top–down, bottom–up, language modality, and ROI as fixed effects (see *SI Appendix*, Table S1 for all model results). To control for sentence planning and wrap-up effects that were not directly related to syntactic structure building, we additionally included a regressor for sentence onset and one for sentence offset. Word rate, word frequency, and number of syllables significantly predicted an increase in BOLD activity. The effect of modality was also marginally significant, with production having more positive activity than comprehension. The effect of modality did not interact with the effect of ROI.

**Fig. 3.**
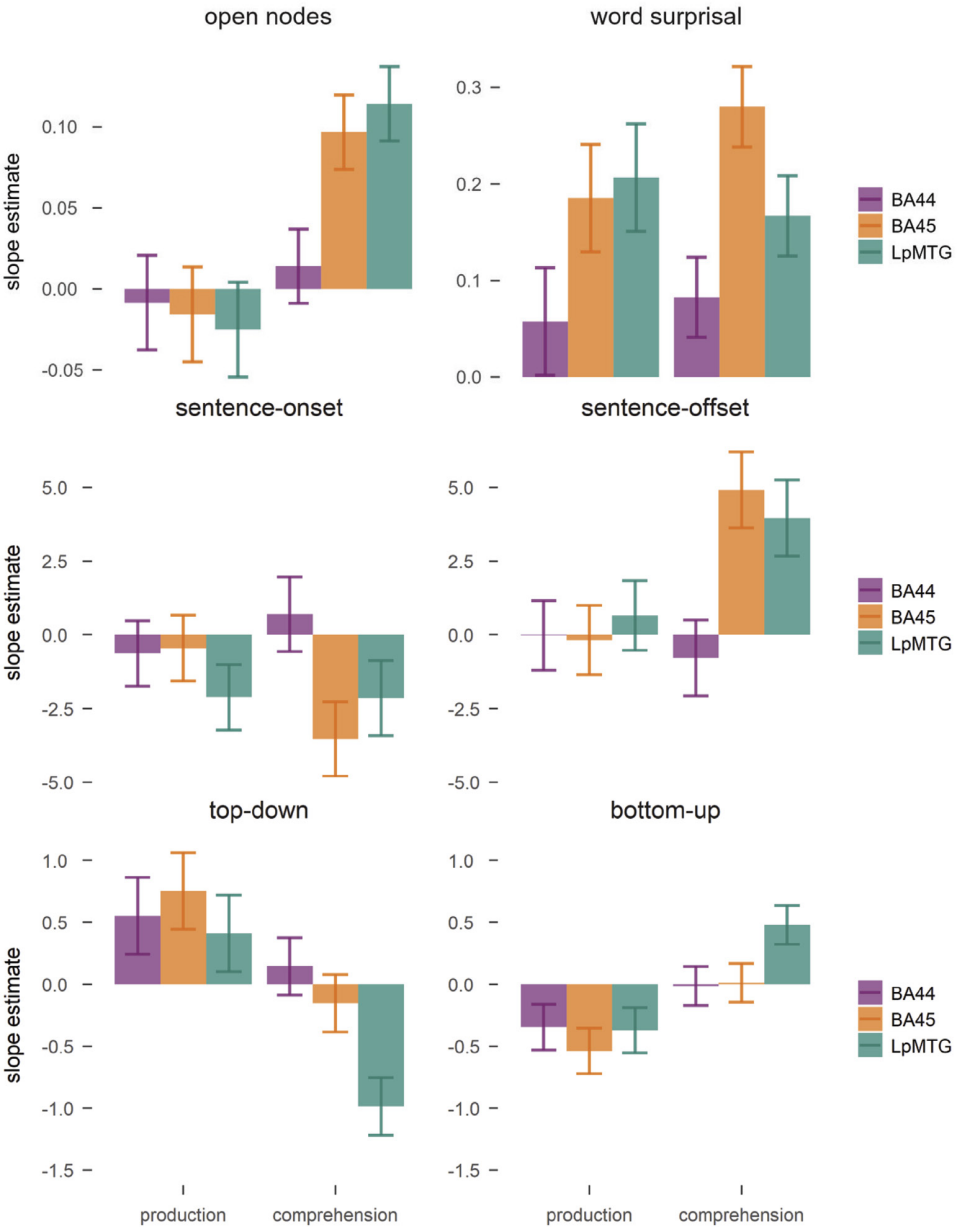
Beta estimates for the effect of open nodes, word surprisal, sentence-onset, sentence-offset, the top–down and bottom–up parsers on BOLD activity in the ROI. Error bars represent SEM.

Larger word surprisal elicited an increase in BOLD in both modalities (Fig. 3, χ^2^ = 51.9, *P* < 0.0001). This effect interacted with ROI (χ^2^ = 17.4, *P* = 0.0002) since BA44 responded significantly less to surprisal than BA45 and LpMTG (pairwise estimates > 0.1, *P* < 0.02) in both modalities. Open nodes also had a significant effect on BOLD activity (Fig. 3, χ^2^ = 8.5, *P* = 0.0035). The effect interacted with modality and ROI (χ^2^ = 12.04, *P* < 0.003). It was significant only in comprehension in BA45 and LpMTG (estimates > 0.99, *P* < 0.001), while the estimates approached zero in all ROIs in production. Open nodes track the number of nodes to be kept in working memory until they can be integrated. It thus seems that the amount of structure that needs to be kept in working memory to be confirmed with the input leads to a brain activity increase in comprehension, but not in production.

Sentence onset and offset predicted the largest variation in brain activity, especially in comprehension (Fig. 3). The results indicated a main effect for sentence onset (χ^2^ = 7.5, *P* < 0.007) and for sentence offset (χ^2^ = 5.7, *P* = 0.017), a marginally significant interaction between sentence-offset and ROI (χ^2^ = 5.7, *P* = 0.056), an interaction between sentence-offset and modality (χ^2^ = 6.4, *P* = 0.01), and a three-way interaction between ROI, modality and sentence-offset (χ^2^ = 6.1, *P* < 0.05). Sentence-onset was related to a decrease in activity overall (estimate = −1.4, t = −2.8), while sentence-offset was related to an increase in activity (estimate = 1.4, t = 2.6) in comprehension (comprehension–production: estimate = 2.5, *P* = 0.011), especially in BA45 and the LpMTG (difference estimates: estimates > 4.7, *p*s < 0.023). Therefore, these results suggest that neural activity in these regions tracks sentence boundaries in comprehension, while production seems less sensitive to sentence boundaries.

We next determined whether incremental metrics of phrase-structure building significantly predicted brain-activity in BA44, BA45, and LpMTG (Fig. 3). Both top–down and bottom–up parsers added significant contributions to the model, in interaction with modality and ROI (three-way interaction for top–down, χ^2^ = 6.6, *P* = 0.036; interaction between modality and bottom–up, χ^2^ = 11.1, *P* < 0.001; interaction between ROI and bottom–up, χ^2^ = 5.9, *P* = 0.052). Anticipatory top–down node counts predicted a significant increase in activity in production relative to comprehension (difference estimate = 0.9, *P* = 0.004*)*. The response to top–down node counts in comprehension was negative, and significantly lower in the LpMTG than in BA44 and BA45 (difference estimates > 0.83, SE = 0.25, *p*s < 0.003), while there were no significant differences among ROIs in production (difference estimates < 0.35, SE = 0.29, *p*s > 0.47). Integratory bottom–up node counts yielded an opposite pattern of results. Larger bottom–up counts led to a significantly lower response in production than comprehension (difference estimate = 0.58, *P* = 0.0009). Again, ROIs responded differently to bottom–up counts across modalities. In comprehension, the strongest response was in LpMTG (difference estimates > 0.47, SE = 0.19, *p*s < 0.043), while in production the responses were negative and not significantly different among ROIs (difference estimates < 0.19, SE = 0.2, *p*s > 0.6). Therefore, activity in all ROIs was related to structure-building in production, while only the LpMTG tracked syntactic structure in comprehension, with opposite dynamics than in production. Activity in BA45 was better predicted by phrase-structure building in production, while in comprehension it was strongly influenced by sentence boundaries. An anonymous reviewer inquired about the responses of additional regions (LATL, RATL, LIPL, LMFG) for comparability with previous comprehension studies (27, 28, 30). To expand our theoretically informed analysis, we explored the responses of these additional regions in *SI Appendix*, Fig. S3.

The parsers thus revealed marked differences between language production and comprehension. Anticipatory node counts led to an increase in neural activity during production, but decreased activity during comprehension. This suggests that during production syntactic structure building dominated at phrase opening. The decrease in activity predicted by the bottom–up parser during production suggests that, at phrase closing, syntactic processing load was reduced. In comprehension, instead, the neural activity increase for bottom–up node counts suggests that syntactic structure building dominated at phrase-closing and was reduced at phrase-opening, when the top–down parser predicted a decrease in activity. Overall, neural activity increased with syntactic structure building in both modalities, but critically with different temporal profiles across the sentences in each modality. Syntactic structure building was seen to elicit an increase in neural activity at phrase-opening in production and at phrase-closing in comprehension.

### Phrase-Structure Building in Production Proceeds in a Highly Incremental Fashion.

The parsing strategies mentioned so far were developed in the context of comprehension. This is problematic because linguistic operations have been assigned *at the time* a word was said. This is a reasonable assumption in comprehension, where processing must follow the input to some extent. However, in production, once a word is articulated, the associated grammatical and lexical encoding will have already taken place (34, 57). We thus explored two production-specific node building strategies that might better account for the timing of syntactic encoding in production: an *early top–down* model and a *chunked* model, both modified from the top–down strategy that was seen to better model neural activity in production. In both, syntactic structure related to a word was assumed to be built at the latest when the previous word was articulated. However, the two strategies made different predictions about the incrementality of structure building.

The *early top–down* model predicted structure building to occur as the previous word was uttered (i.e., at each word we counted the nodes associated with the following word; see *Methods* for more details, Fig. 1*C*). This strategy leads to an equally incremental node building strategy as the original top–down strategy, but, critically, builds nodes earlier, more in line with theories of word production (57, 58). While more fitting for production in terms of timing, this view presupposes a highly incremental syntactic encoder that builds nodes associated to each word in incremental steps, without anticipating a verb phrase when the grammatical subject of the sentence is said.

Studies of sentence planning in production, however, have long debated whether planning is linearly or hierarchically incremental, that is, whether the structure is built from each concept separately or from the relations between concepts (59). Hierarchical models of sentence production consider the verb to be the central node for the syntactic structure (34, 35), suggesting that planning proceeds less incrementally. We thus explored whether a less incremental parser would better account for brain activity than a word-by-word incremental parser. We developed a node building strategy that counted all the nodes between words that were identified as heads according to dependency parsing (see *Methods* for more details, Fig. 1*D* and *SI Appendix*, Fig. S1). This *chunked* strategy predicts chunks of syntactic processing to happen at focal points, in a less incremental way. This approach, by combining dependency and constituent structures, is similar to existing syntactic generators that use a top–down strategy to identify focal points (“semantic heads”) and build nodes up to that point in a bottom–up fashion (40, 60). This generation strategy, called “head-driven generation,” diverges from the parsing strategies introduced above by focusing on the production problem of having to generate a syntactic tree from a semantic structure, rather than a sequence of already identified lexical items.

We compared the initial top–down parser used in the previous analyses with the *early top–down model* and the *chunked model* by fitting three linear mixed models to the production data, each with one of these different predictors of phrase-structure building. The *early top–down* model led to the best model fit [as measured with the Akaike information criterion (AIC), lower values indicate better fit: *early top–down*, 170,803.9; *top–down*, 170,821.3; *chunked*, 170,837.5, *SI Appendix*, Tables S2–S4].

**Fig. 4.**
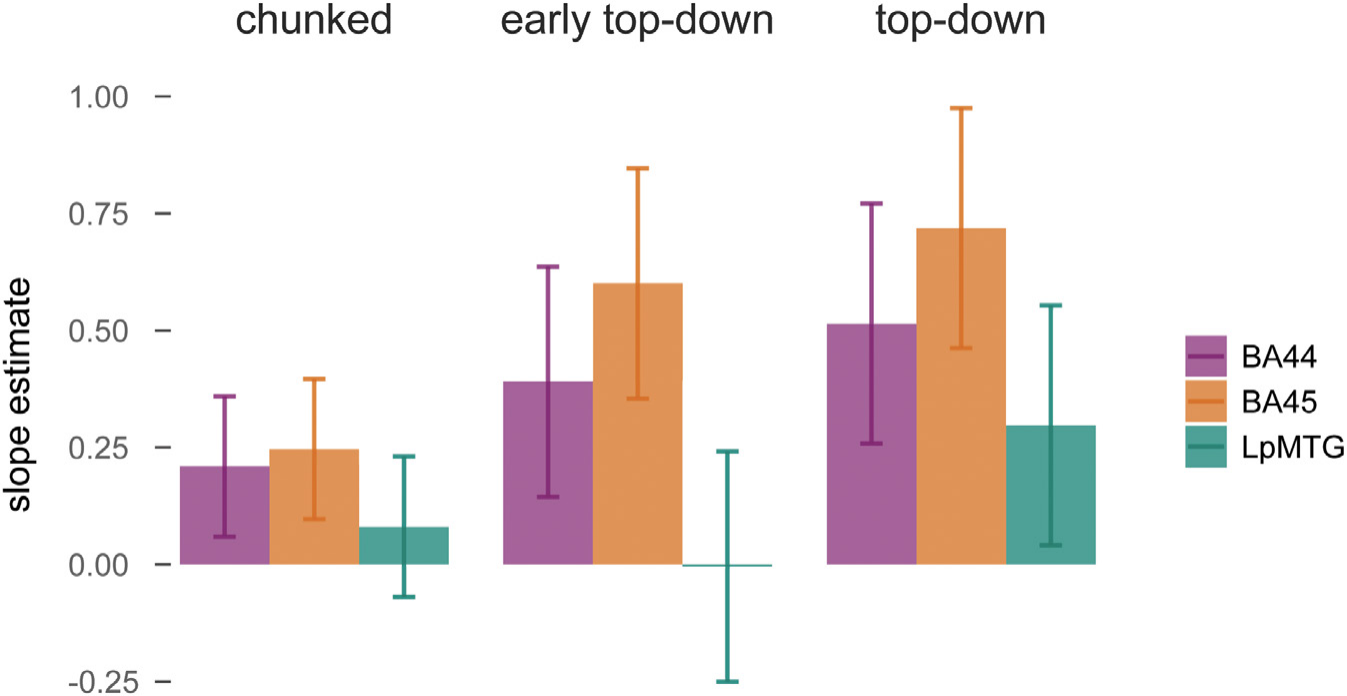
Beta estimates of the effect of each predictor of phrase-structure building in production on BOLD activity in the ROI. Error bars represent SEM. The early top–down model led to the best model fit (AIC, lower values indicate better fit): early top–down, 170803.9; top–down, 170821.3; chunked, 170837.5.

The *top–down* model predicted an overall increase in BOLD (*top–down*, χ^2^ = 7.5, *P* < 0.007), while the *chunked* predictor was not significant (*chunked*, χ^2^ = 2.6, *P* = 0.109). The *early top–down* main effect was not significant (χ^2^ = 2.7, *P* = 0.1), but it interacted with ROI (χ^2^ = 6.2, *P* < 0.05) (Fig. 4). In particular, *early top–down* counts predicted an increase in BA45 (estimate = 0.44, SE = 0.18, *P* = 0.015), while the effect was absent in LpMTG (estimate = 0.003, SE = 0.18, *P* > 0.98). These results were confirmed by likelihood ratio tests of a full model that included all three predictors. We found a significant contribution of both the *top–down* predictor (χ^2^ = 6.17, *P* = 0.013) and of the interaction of *early top–down* with ROI (χ^2^ = 6.42, *P* = 0.011). The involvement of the LpMTG thus decreased when phrase-structure building operations were posited to take place earlier, suggesting that the LpMTG responded to node counts later than the LIFG (see *SI Appendix* for converging evidence on the latency of the response based on analysis of the temporal derivative, *SI Appendix*, Fig. S2). The LIFG instead responded to structure building operations before word onset. The less incremental model of structure building instead did not model brain activity well, providing evidence against the need to plan the structure up to the verb at the start of the sentence. This pattern of results, therefore, indicates that, during production, phrase-structure building operations preferentially took place shortly before word onset in the LIFG in a highly incremental fashion. An analysis on the pausing patterns throughout the speech additionally revealed that top–down node counts affected pause length before word articulation, providing converging evidence for phrase-structure building to happen before word onset in production (see *SI Appendix*, Fig. S4 for the analysis on pause length and word duration).

## Discussion

In the first study to investigate the neural correlates of syntactic processing during spontaneous production, we modeled incremental phrase-structure building with probabilistic parsers and used them to predict brain activity in BA44, BA45, and LpMTG. We found that phrase-structure operations successfully predicted brain activity during naturalistic production and comprehension. A central finding was that the timing of phrase-structure operations differed strongly between production and comprehension. The results suggest that phrase-structure building occurs in an integratory manner in comprehension. Phrase-structure building was instead markedly anticipatory and incremental in production (occurring predominantly before word onset), as evidenced by anticipatory parser operations predicting pause length before each word during speech, and by incremental production parsers that best modeled the production data.

Therefore, parser strategies that have been linked to neural activity in relation to syntactic structure building during sentence comprehension were found here to successfully predict neural activity also during sentence production. This suggests that the syntactic structure built by the parser approximates syntactic processing load suitably in both production and comprehension, and is consistent with sentence production and comprehension interfacing with the same syntactic representations (23, 25). The comparison of top–down and bottom–up parsers highlighted critical differences in the timing of structure building between modalities. Syntactic processing elicited BOLD activity increases in both production and comprehension, but critically the temporal profiles of brain activity diverged across modalities. Superficially, this discrepancy highlights inherent processing differences between language production and comprehension. In production, structure building can proceed by establishing the upcoming structure before words are uttered, which was confirmed by the better fit of the *early top–down parser* with neural activity, as well as by the longer pauses associated with larger numbers of top–down parsing operations. In comprehension, instead, phrase-structure building proceeded in a more integratory manner, whereby the brain waits for the input before committing to a syntactic structure. These results fit with previously obtained evidence on BOLD timing sensitivity to structure complexity and modality, where BOLD peaked earlier with more complex structures in production but later in comprehension, relative to easier structures (12, 46) (see refs. 61 and 62 for converging evidence on production and comprehension dynamics of composition in magneto-encephalography (MEG)). Thus, the present results converge with previous controlled experiments in showing early syntactic encoding in production relative to later encoding in comprehension (12). This is likely due to different processing dynamics in production and comprehension, which have opposite inputs, outputs, and mappings between linguistic levels (17).

Different processing dynamics, however, might be only a symptom of different processing contexts, rather than different processing algorithms between production and comprehension. During syntactic parsing in comprehension, the listener has to resolve structural ambiguity in the input (hypothesis management (63)). Instead, during syntactic encoding in production, the speaker has to make structural decisions to encode the message. Therefore, although the context is different, in both syntactic parsing and encoding the computational goal is to select a structural representation consistent with the input (39). Similarly, Kempen (64) argues that the processes of grammatical encoding in production and parsing in comprehension can form a single processing mechanism used for constructing syntactic structure. The differences between parsing and encoding are due to different processing contexts, where in production lexico-syntactic information is extracted from conceptual structure, while in comprehension the lexico-syntactic information is derived from word strings. Under this assumption, the different dynamics between modalities suggest that the mapping between the semantic context and lexico-syntactic information can happen earlier in production, leading to anticipatory structure building. Instead, in comprehension structure building follows the mapping from sound sequences to lexico-syntactic items. Therefore, diverging dynamics of structure building do not necessarily imply separate structure building processes during production and comprehension.

Interestingly, there were some regional differences in the sensitivity to syntactic predictors in each modality. In particular, in comprehension syntactic processing was related to neural activity only in the LpMTG. The activity of BA45 was instead explained exclusively by sentence boundaries. This dissociation may be explained by BA45 being linked to sentence-level processing taking place at the end of sentences in a way that is not linearly related with the number of nodes. The LpMTG instead seemed to track both sentence wrap-up effects and node-related syntactic load. In production, there was no significant difference between ROIs in how they responded to syntactic operations. However, the results of the production-only parsers and an analysis of the temporal derivative suggest that the LpMTG was more active in production at later latencies. Instead, none of the ROIs were sensitive to sentence boundaries in production, possibly indicating that sentence planning was a more continuous process.

These regional differences in the pattern of responses across modalities may be reconciled with a shared processing account by suggesting that the LpMTG was involved in lexical–syntactic retrieval, while the LIFG was involved in sentence-level processing that was not purely syntactic. In production, as suggested by the top–down parser, the mapping between semantic and syntactic structure may have been supported by the LIFG, with sensitivity to the amount of structure building, engaging the LpMTG at later timescales for lexical–syntactic retrieval. In comprehension, as indicated by the bottom–up parser, lexical–syntactic retrieval in the LpMTG may have preceded sentence-level processing and have appeared at canonical hemodynamic response function (HRF) delays, followed by unification in BA45 at sentence offsets (41). Finally, the evidence for a different latency in the response of the LpMTG and LIFG during production suggests that inconsistencies among studies in the regional patterns of responses may be due to differences in the activation latencies of these regions relative to sentence-level manipulations. The temporal derivative and production parser analyses allowed us to uncover the evidence for the later involvement of LpMTG in production.

It should be noted that these results only outline coarse processing dynamics, given the low temporal resolution of the BOLD signal, and that they do not aim to faithfully model all processes going on during speaking and listening. For example, these parsers are perfect “oracles,” meaning that they always posit phrase-structure building operations for the final structure, ignoring potential ambiguities in the input (52). Recent evidence has shown that modeling syntactic ambiguity improves the fit with brain activity (28). In addition, there is substantial evidence that comprehension is sensitive to the predictability of the input, such that some amount of anticipatory syntactic processing is expected in comprehension as well (5, 65–67). Indeed, Brennan et al. (27) found a positive relationship between top–down operations, syntactic surprisal and BOLD activity in comprehension. Similarly, Coopmans (68) found that a top–down parser best modeled brain activity during comprehension in MEG. Nelson et al. (30) instead found bottom–up counts to better model brain activity (measured with electrocorticography) than top–down counts for the comprehension of single sentences. It is possible that different characteristics of the speech input led to this difference between studies. In our case, the input was spontaneous speech that also included disfluencies and corrections, while Brennan et al.’s and Coopmans’s linguistic input were audiobook stories. There is evidence that lexical predictions can be influenced by reading strategies (69). It might have been easier to anticipate the structure in the “cleaner” audiobook story than in the spontaneous recall of an unfamiliar story. The reduced contextual information available in Nelson et al. (30) may also have led to a reduction in anticipatory syntactic processing. Future studies with naturalistic comprehension will need to clarify to what extent the nature of the input determines the strength of anticipatory vs. integratory syntactic structure building.

Returning to parser-specific modeling of syntactic processing, the parsers discussed so far have usually been discussed in the context of syntactic processing specifically in comprehension (52). Here we also explored modifications of these parsers that were inspired by psycholinguistic findings about syntactic processing in production (17). In production, syntactic processing is thought to happen before word articulation (34, 35, 57). There are different views on whether lexical access guides the structure, or whether the structure encoding the relations between concepts guides the order of lexical access (59). While the evidence provides mixed support for both accounts, suggesting that syntactic encoding is flexible and variable (70–72), several proposals identify the verb as a central node in sentence planning, suggesting that the syntactic structure until the verb needs to be computed before speech onset (35, 73, 74). Cross-linguistic evidence even suggests that in some languages some level of planning happens during the previous sentence (75). By taking advantage of brain activity as an index of processing dynamics, we compared more and less incremental models of sentence planning with two parser models that made different predictions on the temporal unfolding of syntactic structure.

An incremental parser that is more anticipatory than the original top–down parser led to the best model fit, suggesting that structure building proceeds before word articulation. This was also confirmed by converging results on longer pauses before words associated with more phrase-structure building operations, in line with previous behavioral evidence linking pausing patterns in speech with syntactic complexity (76). A less incremental parser that always plans the structure for a few chunks of words at a time provided the worst fit for brain activity. These results suggest that a highly incremental parser may be the more standard planning strategy in production, and that the structure up to the verb does not need to be planned at the start of the sentence. As a note of caution, it is possible that by modeling the *chunked* parser differently, we may have found better fit for this model. In particular, what our results show is that the structure is not planned early and in larger chunks. Modeling word accessibility as well in the parsing strategies to account for variability in incrementality may prove nonetheless to be beneficial. Additionally, future studies may explore strategies that include the semantic structure of the sentence in the structure building process as suggested by the generation literature (40) or word-by-word predictors derived from generators paired with parsers in a way that affords direct comparability between production and comprehension (77).

The finding of highly incremental structure building partly contrasts with previous behavioral results that found verb access in English before speech onset or at least before the production of an internal argument (e.g., refs. 73 and 78). It remains open, though, whether verb access requires the respective structure building to be completed at the same time. It is possible that the verb is accessed early but the structure is not built until after producing the subject. By investigating cross-linguistic differences in the incrementality of structure building, we may be able to understand to what extent the strong incrementality found here also applies to other languages and is directly linked to the latency of verb access found in behavioral studies. In comprehension, this approach highlighted differences in structure-building preferences between Dutch and English, as preference for a top–down strategy was found in a comprehension study in Dutch, relative to the better fit of left-corner parsing in English (52, 68, 79).

We thus provide neuroimaging evidence addressing the long-standing debate on the incrementality of sentence planning. This approach could contribute to the understanding of the dynamics of sentence planning, by developing models that take into account the variability of each sentence, for example by modeling longer planning scopes only when the verb follows an internal argument (74), or depending on word accessibility (71, 72). The approach developed here also has the potential to uncover differences in the incrementality of structure building across languages using a more naturalistic paradigm.

Finally, previous studies found modality differences in the sensitivity of neural responses to syntactic processing (12, 50). In particular, syntactic processing led to stronger responses in production than comprehension. This difference could have been observed either due to task-related effects or due to modality-inherent differences, such as a stronger need in production to fully compute the syntactic structure to be able to speak correctly, in contrast to good-enough processing in comprehension (36, 80, 81). While we could not directly address this question with modality as a between-subject variable, the results indicate that the different modality load on syntactic processing found in previous studies may in effect be task-related. In this study, syntactic structure building elicited a neural activity increase that was quantitatively similar across ROIs in both modalities, although with different dynamics. This finding is consistent with the view that, in contexts where production is spontaneous and unconstrained by artificial tasks, and where comprehension is meaningful and as a consequence more engaging, syntactic parsing and encoding may have a similar load on brain activity.

Importantly, with this study, we demonstrated the feasibility and benefits of studying production with spontaneous speech. The costs associated with spontaneous production, such as increased variability and disfluencies of the linguistic signal, increased motion artifacts in fMRI and the slow temporal resolution, are outweighed by the many advantages. Spontaneous production yields a larger amount of data than controlled tasks. This is the case especially in behavioral analysis but also with fMRI, provided the speech samples are of sufficient length. In addition, with spontaneous speech, the artificiality of the task is largely reduced. Although speaking in monologue is not as common as dialogue, it is much more ecologically valid than speaking following careful instructions with limited acceptable speech output. In addition, the probability distributions of linguistic inputs and outputs are preserved in spontaneous contexts, in contrast with many experiments (82). Finally, neuroimaging studies on spontaneous production allow for potentially new insight into production questions that have been so far mostly addressed with psycholinguistic studies. One limitation of this study is that production and comprehension processes were studied in different participants. Future studies specifically designed to address these questions, with modality as a within-subject variable and a larger sample, will have to confirm the present results. Importantly, the current study shows that studying spontaneous production with fMRI is feasible. In addition, with this design, the task requirements across production and comprehension were better matched than in previous studies addressing production and comprehension differences (12, 50).

In summary, we showed that spontaneous production can be used to study the neural correlates of linguistic processing, providing very rich data that can be directly linked to behavior with the analysis of pause length and word durations. We found that syntactic structure building engages inferior frontal and posterior temporal regions in production and comprehension with diverging dynamics. Phrase-structure building was anticipatory in production but integratory in comprehension. Finally, we provided neural evidence for incremental models of syntactic encoding in production using production-specific parsers. These findings demonstrate the feasibility of studying spontaneous production and begin to uncover the dynamics of structure building in speaking and listening.

## Methods

### Data Acquisition and Preprocessing.

#### Production data.

The production data used were collected by Chen et al. (55, 83) and made available on OpenNeuro (https://openneuro.org/datasets/ds001132/versions/1.0.0), after participants provided informed written consent before the start of the study in accordance with experimental procedures approved by the Princeton University Institutional Review Board. In this experiment, participants watched an episode of the BBC television series *Sherlock* and then recalled what happened in the episode. Data were originally collected for 22 right-handed native English participants (10 female, ages 18 to 26, mean age 20.8). Data for five participants were not shared, since they were excluded due to excessive head motion (2 participants), because recall was shorter than 10 min (two participants) or for falling asleep during the movie (one participant). Data for one participant were not shared because of missing data at the end of the movie scan, which left us with 16 participants for the current analysis. Speaking led to an average framewise displacement of 0.32 (average per participant, range = 0.13 to 0.54), which was higher than the average in the comprehension data (0.22, range = 0.08 to 0.42) but was corrected for with noise regression (see fMRI data preprocessing in *SI Appendix* for more details).

Participants watched the first 50 min of the first episode of the BBC TV series *Sherlock*, after confirming that they had not watched any episode of *Sherlock* before. Participants were told they would be asked to verbally describe what they had seen. After watching the episode, they were immediately instructed “to describe what they recalled of the movie in as much detail as they could, to try to recount events in the original order they were viewed in, and to speak for at least 10 min if possible but that longer was better. They were told that completeness and detail were more important than temporal order, and that if at any point they realized they had missed something, to return to it. Participants were then allowed to speak for as long as they wished, and verbally indicated when they were finished (for example, “I'm done”). During this session, they were presented with a static black screen with a central white dot (but were not asked to, and did not, fixate) (83). Their speech was recorded in the scanner with an MRI-compatible microphone.

We also used a second production scan for one of these participants, who also recalled an episode of BBC TV series *Merlin*, as part of the data collected and released by Zadbood et al. (84). This speech sample was used as audio stimulus for part of the Comprehension data (see below). The procedure and acquisition were the same. Therefore, in total, we used 17 speech samples from 16 participants, since one participant recalled both *Sherlock* and *Merlin*. The 17 recalls were 10 to 45 min (mean = 22 min, SD = 8.8 min), including on average 2,874 words (range = 1,666 to 6,230, SD = 1,299).

#### Comprehension data.

For the comprehension data, we used the data shared by Zadbood et al. (56, 84) on OpenNeuro (https://openneuro.org/datasets/ds001110/versions/00003), after participants provided informed written consent before the start of the study in accordance with experimental procedures approved by the Princeton University Institutional Review Board. In this experiment, participants listened to an audio recording of the recall of one production participant from the production data (see above) about either *Merlin* or *Sherlock*. They also watched an episode of the BBC TV series *Merlin* or *Sherlock* (note that they listened to and watched different stories), which was not analyzed here. Audio recordings were obtained from a participant that watched and recounted the two movies, here analyzed as part of the production data. In this dataset, 52 right-handed native English speakers (age 18 to 45) were scanned. Fifteen participants were excluded because of head motion (n = 4), for falling asleep (n = 4), due to poor memory (n = 5), for having seen the movie before (n = 2). This resulted in 36 shared and analyzed participants, 18 that listened to the Merlin recall, and 18 that listened to the Sherlock recall. The audio recall for Merlin was 14.7 min long and included 2,141 words. The audio recall for Sherlock was 17.5 min long and included 2,468 words.

Although the production and comprehension datasets were collected as part of separate experiments, the datasets were acquired with the same scanning parameters (*SI Appendix*). The task was as similar between modalities as differences between production and comprehension allow. In particular, in production the linguistic output was the spoken recall of a TV episode, and in comprehension the linguistic *input* was the recall of a TV episode (produced by one of the production participants).

### Incremental Complexity Metrics.

#### Syntactic tree extraction with the Stanford parser.

First, we extracted the constituent structure of each sentence with a probabilistic context-free phrase-structure grammar. We used the Stanford parser with CoreNLP in Python 3 via the Natural Language Toolkit package (51, 85). The transcript provided in the shared dataset was divided in what we considered independent sentences. Since the production was very spontaneous and unconstrained, sentence boundaries were not objective and self-evident as they are in text. In speech, the boundaries can depend on the syntactic structure of the sentence, but also on pausing patterns and disfluencies. For example, coordinated clauses may be considered one single sentence or divided into two separate sentences based on pause lengths. Also, some sentences extend over 30 words or more, with many embedded phrases. Participants, however, do not appear to always keep in working memory the original syntactic structure, which is revealed by their disfluencies and corrections throughout long sentences. In particular, boundaries could be set to track the syntactic structure, also including hesitations and corrections within its boundaries, or to track speech patterns and “reset” every time there is a disfluency. After extensive exposure to the transcripts, it became clear that shorter boundaries better reflect the planning chunks followed by speakers in these monologues without audience feedback, but it is to be determined if different approaches work better in other contexts. For example, the following text could be considered a single sentence: “I believe at this point we're shown this string of three successive suicides which are immediately suspicious to the viewer because they're they have their origins in these mundane situations.” However, there was a pause of 2.6 s after “because.” The sentence was therefore divided into: “I believe at this point we're shown this string of three successive suicides which are immediately suspicious to the viewer because. They're. They have their origins in these mundane situations.” Now the false start is a sentence by itself, which ensures that syntactic processing taking place at that time is captured, but it is less likely to affect and possibly confuse the full sentence parse. The sentence boundary at “because” is not problematic for the parser: the node counts are equivalent, with the difference that the sentence starting at “they” is not attached to the previous part. This means that at “situations” the bottom–up counts refer all the way back to “they” but not “I believe.” This is not necessarily problematic, given the long pause between “because” and “they,” which makes it unlikely that the speaker was referring back to the original S node at “situations.” It should be noted that an initial analysis was run on longer sentences, which perhaps better tracked the overarching syntactic structure but did not optimally reflect the planning processes of participants. The results were similar with both sentence boundaries approaches, but the disfluency-informed approach to sentence length was less noisy. The average number of sentences per participant was 307 (±168) formed by 9.4 (±1.2) words for the disfluency-informed approach, and 196 (±115) sentences formed by 14.9 (±2.1) words for the initial longer sentence approach.

Since the Stanford parser was trained on newspaper articles, we performed a validation procedure to make sure that it was able to appropriately capture the syntactic structure of spontaneous and disfluent speech. We randomly selected 10 sentences per participant (170 sentences and 1,434 words out of the 3,328 sentences and 47,153 words produced in total) and manually corrected the output of the parser. From the selected set, 39 out of the 170 reviewed sentences included at least one error. Errors most often stemmed from a wrong attachment or wrong part-of-speech marking and were thus not directly linked to disfluencies. Only four of these errors were due to the presence of filled hesitations such as “like” or discourse markers like “you know.” We determined parser accuracy as the correlation between the parser counts from the original parse and the reviewed parser counts. The correlation between the adjusted top–down counts and the uncorrected top–down counts was 0.92. The bottom–up correlation was 0.93. We expect the correlation would effectively be higher after convolution with the HRF, since the parsing errors usually resulted in nodes being assigned a few words earlier or later, which often fall within the same TR. (It was not interesting to convolve the corrected parser counts with the HRF, since the corrected sentences were not contiguous in time.) We thus considered the performance of the Stanford parser for spontaneous speech appropriate for our purposes.

#### Phrase-structure parsing.

Following sentence structure extraction with the Stanford constituent parser, we took a measure of syntactic processing with incremental complexity metrics derived from the number of syntactic nodes that are built with each word. Nodes can be built with different parsing strategies: top–down, bottom–up and left-corner (52). In top–down parsing, nodes are built from the top of the syntactic tree to the terminal node (corresponding to a word). In other words, nodes are counted when phrases are opened. This strategy can lead to the anticipation of nodes that may not always be known to a listener. For example, in the sentence “Mary eats apples daily,” a node accounting for the upcoming presence of “daily” is counted already at the word “eats.” This anticipation is justifiable in production, where the upcoming structure is presumably known to the speaker in advance, but it might reflect unjustifiable prediction in comprehension. Nevertheless, this implementation of a top–down strategy may be successful in accounting for predictive processes in comprehension.

At the other end of the incremental parsing spectrum is bottom–up parsing, according to which nodes are built from the bottom of the syntactic tree (i.e., from the terminal nodes, corresponding to each word) up to the highest *closed* nodes, i.e., nodes where all daughter nodes have already been met. For example, in Fig. 1*B*, the top node in purple (S) cannot be built until its right-branching node VP is built as well, which in this case only happens at the end of the sentence. In other words, bottom–up parsing builds nodes when phrases are closed. This strategy thus predicts increased syntactic processing at the end of clauses and sentences, after all the evidence for the structure is encountered. We expected this parsing strategy to better reflect processing in comprehension than production, because in the latter the structure is presumably already built before the last word is uttered. Neither top–down nor bottom–up parsing strategies fully match human performance (52, 79), but they capture aspects of syntactic processing that are expected to differ across modalities. Finally, left-corner parsing needs less evidence than bottom–up parsing to count nodes, but is not as predictive as top–down parsing. After convolving with the hemodynamic response function, left-corner was highly correlated with the top–down parser (*SI Appendix*, Figs. S5 and S6). Therefore, we decided to only focus on opposite parsing strategies that were most expected to differ between production and comprehension, i.e., top–down and bottom–up.

We also counted the number of nodes that were still open at each word with an *open nodes* measure, similarly to Nelson et al. (30). Open nodes were the number of nodes that were open at each word: This measure tracked the number of nodes that had been opened up to the word and that had not been closed yet, thus providing an index for the number of nodes that need to be kept in working memory until they can be merged in a constituent (30).

#### Production-specific parsing operations.

To account for the timing that is specific to production, we developed two production-specific parsers. An *early top–down* model counts the nodes that are built for the *next* word. At the first word of the sentence, nodes are counted for the first and second words (even though nodes built for the first word would have been built earlier, we preferred this over making assumptions on *when* the nodes would be built before the sentence, which could be varying due to different factors). At the second word, nodes are counted for the third word, etc.

For the less-incremental *chunked* parsing, we selected the heads of each sentence following dependency parsing (see *SI Appendix* for more information on the analysis on dependency parsing). We considered as heads all words that had a dependent relation attached to them (e.g., the verb is head of subject and object). We then counted all nodes (of the same constituent structure used by the other parsers) encountered from the first word up to and including the next head, then from the head up to and including the next head, and so on. Chunked parsing, therefore, builds nodes early on for all the upcoming words that are dependent relations until the next head. For example, at the start of a sentence all the nodes are built for the structure up to and including the verb, usually the first head.

It should be noted that *top–down*, *early top–down*, and *chunked* measures were highly correlated after convolving with the hemodynamic response function (*SI Appendix*, Figs. S5 and S6). To avoid collinearity, instead of comparing them in the same model, we tested models with only one predictor and determined which model provided the best fit (see *Regression analysis* for more details).

#### Word surprisal.

We quantified word surprisal from transformer model GPT-2 (54). We used GPT-2 XL via the TensorFlow implementation provided by HuggingFace’s Transformers package (86). Each word’s probability was based on a context of at least 700 words after the first 700 words of each participant’s recall. Surprisal was calculated as the negative logarithm of the conditional probability of the word based on context. With word surprisal we aimed to control for effects of context on single word processing.

### Behavioral Analysis.

To determine whether these indices of processing complexity had an effect on participants’ speech patterns, we inspected how they affected word duration and pause lengths in all the production recalls. Recordings were not made available with the Production dataset, but word timestamps for each participant’s recall were shared by Janice Chen’s lab available at (87). Onsets and offsets of each word were obtained with *Gentle*. We ran a linear mixed-effects model with *lme4* [version 1.1-26 (88)] in R (version 4.0.3). We used number of syllables, word frequency, word surprisal, top–down, bottom–up and open nodes as predictors for pause length (before the word characterized by each predictor) and word duration. This analysis allowed us to compare neural effects with behavioral patterns of speech.

### fMRI Analysis.

#### Predictor timeseries.

Each word-by-word predictor was mean-centered (except for the word rate predictor, and the sentence-onset and -offset predictors) and convolved with the canonical hemodynamic response function following SPM’s double gamma function as computed in *nilearn*. We thus obtained predictor timeseries temporally resampled to the acquisition TR of 1.5 s, reflecting BOLD increases and decreases following predictor weights time-locked to word onset (Fig. 2*C*).

#### ROI selection.

We selected 3 ROIs that have been associated with syntactic processing in previous studies: two LIFG ROIs, following the distinction between LIFG *pars opercularis* (BA44) and LIFG *pars triangularis* (BA45), and LpMTG. After preprocessing the fMRI data, we selected the ROIs for each participant in their functional space. BA44 and BA45 were extracted following Freesurfer’s label creation with the Destrieux Atlas (89) and resampled to functional space with bbregister. Freesurfer’s MTG ROI is quite long in extension, following the gyrus from very posterior portions to the temporal pole. We therefore extracted this ROI and then masked it with a posterior temporal lobe mask (posterior to Heschl’s gyrus) based on the Harvard-Oxford cortical atlas. Examples of these ROIs in MNI brain can be seen in Fig. 2*E*.

#### Timeseries extraction.

The BOLD timeseries were extracted with NiftiLabelsMasker from *nilearn* (90), after confound regression, from preprocessed data (*SI Appendix*). Framewise displacement, DVARS (derivative of bulk head motion variance over voxels), motion parameters, aCompCor parameters and ICA-AROMA (Independent Component Analysis for Automatic Removal of Motion Artifacts) regressors classified as noise were used for noise regression, to reduce the impact of motion artifacts caused by speaking. The timeseries was extracted from the functional BOLD volumes in functional space as an average of the voxels in each ROI mask.

#### Regression analysis.

To determine to what extent each of these continuous indices of syntactic processing significantly affected brain activity (average BOLD activity in the three ROIs), we used linear mixed-effects models with *lme4* [version 1.1-26 (88)] in R (version 4.0.3). We used a baseline model that included word rate (i.e., a predictor indicating the onset of each word), syllable rate, as an index of articulatory rate, log-transformed word frequency, and word surprisal. All models additionally included modality and ROI as factors. Modality (production vs. comprehension) was contrast-coded with deviation coding. We used Helmert coding for ROI, contrasting LIFG with LpMTG, and the two LIFG *partes* with each other. All other factors were continuous numerical predictors. All models included word surprisal and its interaction with ROI and modality. All models also included by-participant random slopes for syllable rate, frequency, word surprisal, and other factors of interest, excluding by-participant random effects and correlations to allow for convergence and avoid singularity issues. In some cases, we had to exclude the random slopes for one of these factors, but never for the factor of interest in that model. We computed the contribution of factors to the models using *car* [version 3.0-10 (91)], and pairwise comparisons with the package *emmeans* [version 1.6.1 (92)].

The first model determined the contribution of top–down and bottom–up metrics of phrase-structure building to brain activity in the three ROIs and in each modality to a baseline model that included word surprisal and open nodes, as well as sentence onset and offset regressors to account for sentence planning and wrap-up effects that were not related to structure building operations. The sentence onset regressor included a “1” at each first word of each sentence, while the sentence offset regressor a “1” at the last word of each sentence (and 0s for all other words). After convolutions with the HRF, these two regressors were highly correlated with each other, and negatively correlated with the open nodes predictor, which tends to increase throughout the sentence. The interactions of each metric with ROI and modality were also included in the model and the significant contribution of the incremental metric in a region or modality was determined with pairwise comparisons. With this model, we also determined to what extent word surprisal and open nodes affected brain activity in each modality.

We then used three models to ask whether metrics of syntactic processing fine-tuned for production would improve model fit. These metrics are not realistic for syntactic processing in comprehension, so the models only included production data. The baseline models all included word surprisal and bottom–up parser operations, and additionally included *top–down*, or *early top–down*, or *chunked* predictors of phrase structure building and their relative by-participant random slopes. Since the three parsers were highly correlated after convolving with the HRF, we separately fitted three linear models. We compared model fit with the AIC, where more negative values indicate better model fit (93). We additionally tested whether the different predictors added significant contributions to the baseline model with all three syntactic predictors using likelihood ratio tests.

## Supplementary Material

Appendix 01 (PDF)

## Data Availability

Word timestamps with linguistic annotations and the analysis code have been deposited in OSF (DOI 10.17605/OSF.IO/QJMKY). Previously published fMRI data that were used for this study are available on OpenNeuro (https://openneuro.org/datasets/ds001132/versions/1.0.0, https://openneuro.org/datasets/ds001110/versions/​00003) (55, 56).

## Footnotes

^*^Notable exceptions are the studies of Stephens et al. and Silbert et al. (94, 95), who focus on the general process of speaking, rather than on specific features of linguistic processing during production and comprehension.
