# Supplementary material for: Diverging neural dynamics for syntactic structure building in naturalistic speaking and listening
Source: Proc Natl Acad Sci U S A. 2024 Mar 5;121(11):e2310766121. doi: 10.1073/pnas.2310766121 (PMC10945772; doi:10.1073/pnas.2310766121)
Supplement: Supplementary file 1 — Appendix 01 (PDF) [file pnas.2310766121.sapp.pdf]

PNAS

Supporting Information for

Diverging Neural Dynamics for Syntactic Structure Building in Naturalistic Speaking and Listening

Laura Giglio[1,2,*], Markus Ostarek[1], Daniel Sharoh[1,2] and Peter Hagoort[1,2]

[1] Max Planck Institute for Psycholinguistics, Nijmegen, The Netherlands
[2] Donders Institute for Cognition, Brain and Behaviour, Nijmegen, The Netherlands

* Corresponding author: Laura Giglio
Email: laura.giglio@mpi.nl

This PDF file includes:

Supporting text
Figures S1 to S6
Tables S1 to S7
SI References

Dependency parsing

We extracted dependency parsing as an index to perform chunking of each sentence in relational units. We used the dependency parser provided by the Stanford parser via CoreNLP. We identified the heads of the sentence as the words that have a relation attached to them. For example, in the sentence “He’s examining one of the bodies”, “examining” is the first head, followed by “one” and by “bodies” (Fig. S1). These three words are the only ones that have a dependency relation attached to them, while the other words are all dependents of one head. The *chunked* parsing strategy counted the nodes intervening between all heads, to model a less incremental strategy to syntactic structure building, following the idea that speakers plan the structure of a few words at a time (e.g. always planning the structure of the verb at the start of the sentence).

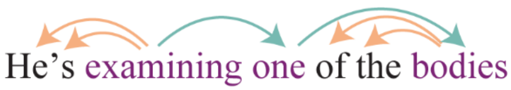

Fig. S1. Dependency parse of the sentence. Left-relations are in orange, right relations in green, heads are in purple. Heads are words on which a dependency relation is attached (i.e. from which an arrow starts).

Temporal derivative

Methods. Since the results of production-specific parsers indicated that the LpMTG may have had a later response to top-down operations than BA45, we looked into how the temporal derivatives of the top-down and bottom-up parsers modelled the data. The temporal derivative of the haemodynamic response function (HRF) is usually used in fMRI analysis to account for small differences in the latency of the BOLD response. An increase in the temporal derivatives means that the BOLD response peaks earlier, while a decrease indicates a later peak. We ran the same linear-mixed effects model used before with the addition of temporal derivatives of all predictors of interest (Table S5).

Results. We found a significant three-way interaction between the top-down derivative, modality and ROI ($\chi^2 = 16.6$, $p < 0.0003$) (Fig. S2); the surprisal derivative, modality and ROI ($\chi^2 = 16.9$, $p < 0.0003$); and the open nodes derivative, modality and ROI ($\chi^2 = 10.7$, $p < 0.005$), and a two-way interaction between the sentence offset derivative and ROI ($\chi^2 = 9.3$, $p < 0.01$). The top-down temporal derivative was significantly positive in BA45 in production (estimate = 0.39, SE = 0.16, $p = 0.02$), indicating an earlier BOLD peak than assumed by the canonical HRF, while it was significantly negative in LpMTG (estimate = -0.33, SE = 0.17, $p < 0.05$), indicating a later peak. It was not significantly different from zero in comprehension, nor did it differ between ROIs. These results suggest that the LpMTG may have been active after BA45 in response to more top-down node counts.

Sentence offset elicited later responses in comprehension in the LpMTG (estimate = 0.6, SE = 0.16, $p = 0.001$), suggesting that the effect of sentence offset was sustained for some time after the end of the sentence. Word surprisal elicited later BOLD peaks in comprehension and earlier BOLD peaks in production, relative to the canonical HRF (difference estimate = 0.8, SE = 0.24, $p = 0.007$). In comprehension, BA45 and LpMTG were both significantly related to a decrease in activity (BA45: estimate = 0.43, SE = 0.16, $p = 0.007$; LpMTG: estimate = 0.61, SE = 0.16, $p = 0.0001$). In production, activity in both BA44 and BA45 increased with the temporal derivative for surprisal (BA44: estimate = 0.49, SE = 0.2, $p = 0.023$; BA45: estimate = 0.96, SE = 0.2, $p < 0.0001$). These results suggest that word surprisal elicited earlier activity increases in production than comprehension, which likely relates to the timing of lexical access (before word onset in production, after word onset in comprehension). The open nodes measure showed earlier BOLD responses in the LpMTG in production (estimate = 1.03, SE = 0.5, $p = 0.035$), and later responses in BA44 and BA45 in production (BA44 estimate = 1.3, SE =0.5, $p = 0.008$; BA45 estiamte = 1.3, SE = 0.5, $p = 0.02$). Again, BA45 and the LpMTG had different BOLD peak latencies, suggesting that BA45 responded earlier to top-down nodes but later to open nodes, while LpMTG responded earlier to open nodes and later to top-down nodes.

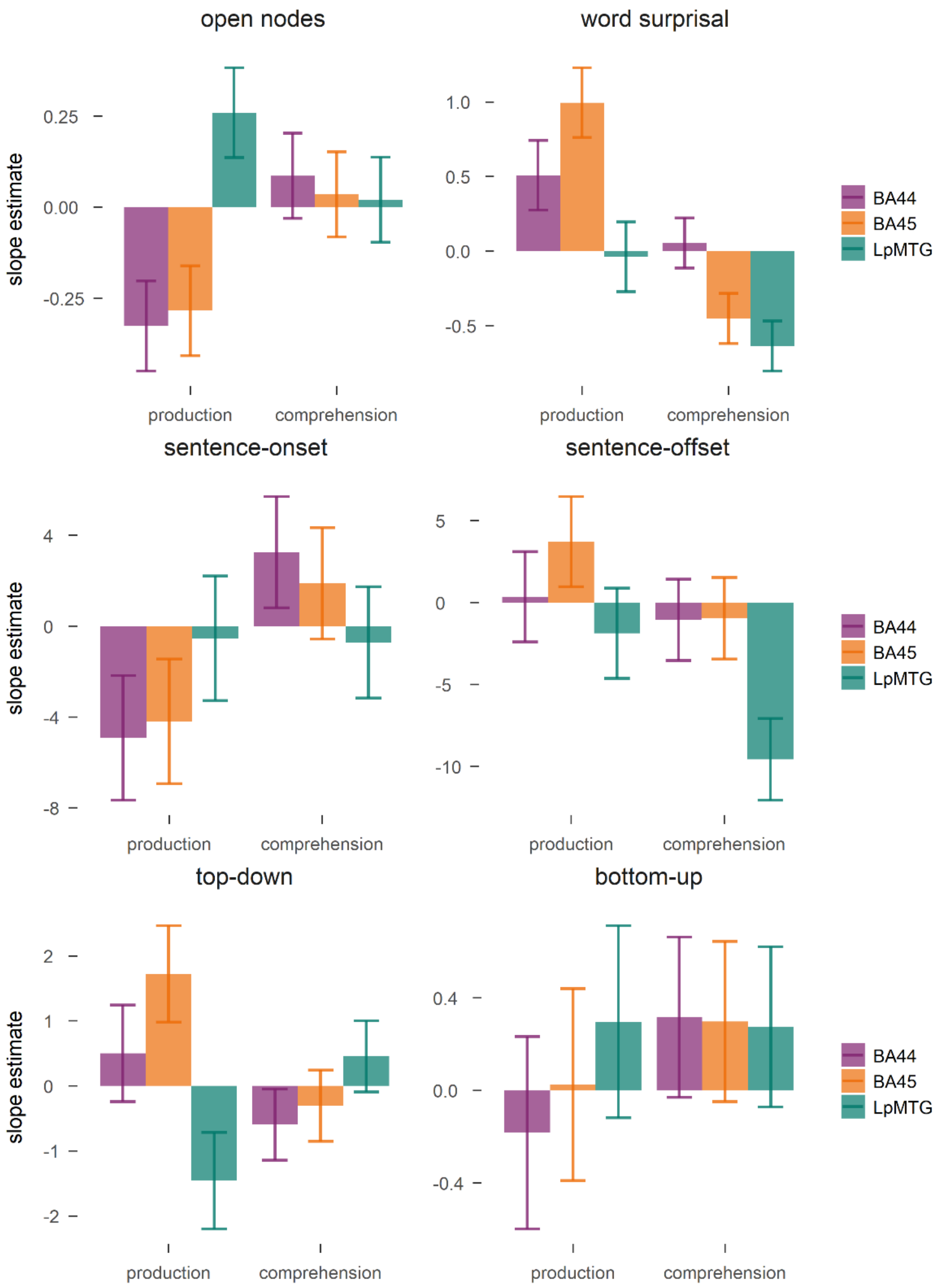

Fig. S2. Beta estimates for the effect of the temporal derivative of each predictor on BOLD activity in the regions of interest. Error bars represent standard error of the mean. Positive estimates indicate an earlier BOLD response, negative estimates indicate a later BOLD response. Note that the y-axis range differs between plots.

Additional ROI analysis

For comparability with previous studies, we ran the same analysis presented in "Distinct dynamics for phrase-structure building in language production vs. comprehension" in a few additional regions often found to respond to language processing, but not known to be strongly involved in syntactic processing: left anterior temporal lobe (LATL), right anterior temporal lobe (RATL), left inferior parietal lobule (LIPL), left middle frontal gyrus (LMFG) (1–3).

Methods. As done for the main regions of interest, we made anatomical masks of these regions using the Harvard Oxford atlas (max probability 25%). The LATL and RATL masks included the labels for the temporal pole, the anterior superior, middle and inferior temporal gyri. The LIPL mask included the left posterior supramarginal gyrus and the angular gyrus. The LMFG included the mask for the left middle frontal gyrus. We then only used the grey matter voxels in functional space for each participant, using freesurfer's aparc.a2009s grey matter mask. We averaged the timeseries for each of these regions and ran the analysis with linear mixed-effects models as specified in the *fMRI analysis* methods section. Instead of using ROI as a fixed effect, now we ran separate analyses for each region (the values reported below are not corrected for the comparison of four ROIs, thus $p < 0.0125$ indicates a significant effect after correction).

Results. BOLD activity in the LATL increased as a function of words ($\chi^2 = 112.5$, $p < 0.0001$), syllables ($\chi^2 = 8.2$, $p < 0.005$), frequency ($\chi^2 = 7.1$, $p < 0.008$), word surprisal ($\chi^2 = 10.2$, $p < 0.002$), top-down parser ($\chi^2 = 8.2$, $p < 0.004$) and sentence offset ($\chi^2 = 16.2$, $p < 0.0001$), and decreased for sentence onset ($\chi^2 = 23.4$, $p < 0.0001$). There was also an interaction between modality and top-down ($\chi^2 = 6.1$, $p < 0.02$), modality and open nodes ($\chi^2 = 16.8$, $p < 0.0001$), and modality and sentence offset ($\chi^2 = 15.3$, $p < 0.0001$). Pairwise comparisons indicated that there was a higher response to top-down node counts in production than comprehension (estimate = 0.6, $p = 0.013$), a higher response to open nodes in comprehension than production (estimate = 0.08, $p < 0.0001$), and a higher response during sentence offsets in comprehension than production (estimate = 5.3, $p = 0.0001$). The results in the LATL, therefore, are similar to the main results in the LpMTG and LIFG.

Activity in the RATL increased as a function of words ($\chi^2 = 67.9$, $p < 0.0001$), syllables ($\chi^2 = 4.2$, $p < 0.05$), top-down parser ($\chi^2 = 8.5$, $p < 0.004$) and sentence offset ($\chi^2 = 7.2$, $p < 0.008$), and decreased for sentence onset ($\chi^2 = 4.4$, $p < 0.04$). There was also an interaction between modality and bottom-up ($\chi^2 = 4.3$, $p < 0.04$), modality and word surprisal ($\chi^2 = 4.2$, $p < 0.04$) and modality and sentence offset ($\chi^2 = 6.1$, $p < 0.02$). Pairwise comparisons indicated that there was a higher response to bottom-up counts in production than comprehension (estimate = 0.5, $p = 0.038$), there was a higher response to word surprisal in comprehension than production (estimate = 0.11, $p = 0.039$), and there was a higher response to sentence offset in comprehension than production (estimate = 3.5, $p = 0.013$). Therefore, the RATL showed a different pattern of responses to the syntactic predictors than the other regions, suggesting that the computations taking place in the right ATL may differ in timing pressures from the left lateralized regions of interest.

BOLD activity in the LIPL increased as a function of words ($\chi^2 = 60.8$, $p < 0.0001$), frequency ($\chi^2 = 16.3$, $p < 0.0001$), word surprisal ($\chi^2 = 14.4$, $p < 0.001$) and decreased for sentence onsets ($\chi^2 = 7.2$, $p < 0.01$). The syntactic predictors had no effect in the LIPL and there was no interaction with modality.

Activity in the LMFG increased as a function of words ($\chi^2 = 22.4$, $p < 0.0001$) and frequency ($\chi^2 = 5.8$, $p < 0.02$). There was an interaction between modality and top-down ($\chi^2 = 4.2$, $p < 0.05$), modality and bottom-up ($\chi^2 = 14.3$, $p < 0.0002$), modality and open nodes ($\chi^2 = 4.2$, $p < 0.05$). Pairwise comparisons indicated that there was a higher response to top-down counts in production than comprehension (estimate = 0.532, p =0.041), a higher response to bottom-up counts to comprehension than production (estimate = 0.64, $p = 0.0002$), and a higher response to open nodes in comprehension than production (estimate = 0.06, $p = 0.042$).

Overall, all regions except for the LIPL showed some sensitivity to syntactic predictors that broadly matched the responses in the LpMTG and LIFG, with differences in the sensitivity to top-down and bottom-up counts between production and comprehension. The response of these regions to the syntactic predictors may have been due to the contribution of these regions in overall sentence-level compositional processes.

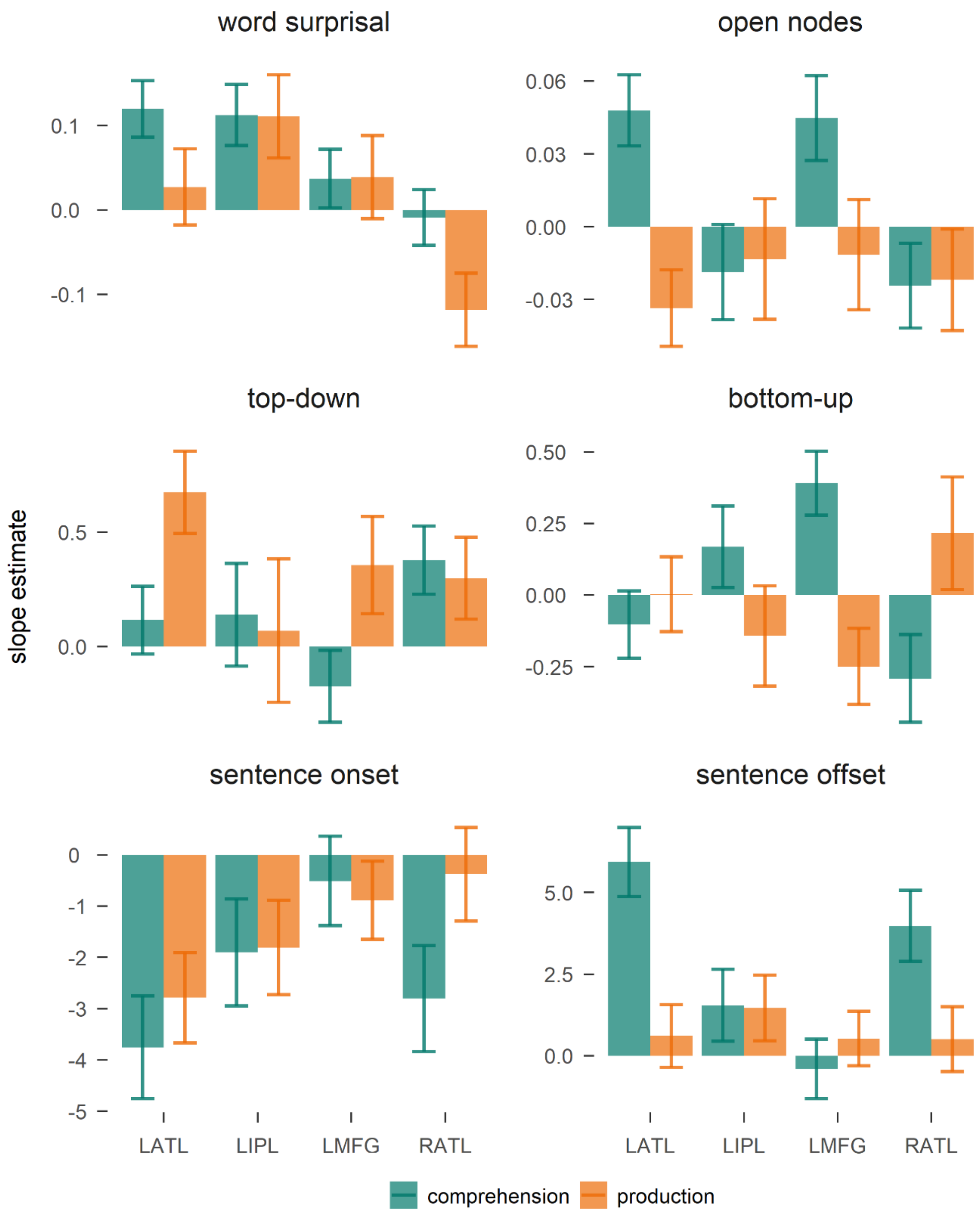

Fig. S3. Beta estimates for the effect of each predictor on BOLD activity in additional regions. Error bars represent standard error of the mean. Note that the y-axis range differs between plots.

Speech fluency

Methods. We analysed whether word and syntactic predictors also explained variance in word duration and in length of pauses before word uttering in the production data. Pauses were defined as the interval between the offset of the previous word and the onset of the current word. We used linear mixed-effects models with pause length or word duration as dependent variables. We used word frequency, word surprisal, number of syllables, top-down, bottom-up and open nodes predictors as fixed effects and as by-participant random slopes (Fig. S3).

Results and discussion. The number of syllables of a word significantly predicted an increase in word duration (unit is seconds, $\beta = 0.08$, SE = 0.004, $t = 22.6$, $\chi^2 = 510.02$, $p < 0.0001$), which was expected, but a decrease in pause length before word articulation ($\beta = -0.02$, SE = 0.004, $t = 4.9$, $\chi^2 = 23.9$, $p < 0.0001$). The shorter pause before articulation of long words is possibly due to the longer time available to plan for later words during uttering of a long word. Higher word frequency instead predicted shorter word duration ($\beta = -0.017$, SE = 0.001, $t = 15.1$, $\chi^2 = 239.8$, $p < 0.0001$), but did not affect pause length ($\beta = 0.0008$). Larger word surprisal increased word duration to a small extent ($\beta = 0.004$, SE = 0.0004, $t = 9.6$, $\chi^2 = 91.9$, $p < 0.0001$), and it had a larger positive effect on pause length ($\beta = 0.02$, SE = 0.002, $t = 10.4$, $\chi^2 = 109.1$, $p < 0.0001$). Less predictable words based on context thus took longer to be initiated and were uttered for a slightly longer time (4 ms), after accounting for their length.

We also determined how predictors of syntactic complexity related to speech fluency. Top-down node counts predicted the largest decrease in word duration ($\beta = -0.045$, SE = 0.002, $t = 20.1$, $\chi^2 = 404.1$, $p < 0.0001$), suggesting that when phrases are opened, information can be conveyed faster, possibly to offload working memory. It also predicted the largest increase in pause length before the word in question is uttered ($\beta = 0.09$, SE = 0.008, $t = 10.9$, $\chi^2 = 119.7$, $p < 0.0001$) suggesting that grammatical encoding related to a word is performed before word articulation, and that nodes are built in an anticipatory way. Bottom-up parser operations predicted an opposite pattern. Larger bottom-up counts increased word duration ($\beta = 0.012$, SE = 0.0009, $t = 12.6$, $\chi^2 = 159.5$, $p < 0.0001$), but decreased pause length ($\beta = 0.021$, SE = 0.002, $t = 11.6$, $\chi^2 = 135.7$, $p < 0.0001$). The shorter pauses suggest that at phrase closing the structure is already computed. Finally, open nodes predicted a significant but very small decrease in word duration ($\beta = -0.002$, SE = 0.0004, $t = 5.3$, $\chi^2 = 28.5$, $p < 0.0001$), and a larger decrease in pause length ($\beta = -0.024$, SE = 0.002, $t = 14.6$, $\chi^2 = 213.9$, $p < 0.0001$), suggesting easier processing the further along in a sentence. In line with the neuroimaging results, this pattern of results suggests that phrase-structure building happens before word articulation and at phrase-opening, with a decrease in pauses the further along in the sentence.

This was the first study to show an increase in neural activity for words associated with higher surprisal, not only in comprehension but also in production. Many studies showed sensitivity of brain activity to surprisal in language comprehension computed with several models (4, 5). The neural results are in line with the behavioural results that show an increase in pause length before less probable words and a small increase in their duration, as found previously (6). The results thus converge in demonstrating the sensitivity of the production system to the statistical probabilities of the linguistic input and output, both in behavioural and neural patterns. This finding is in line with accounts of efficient language production that propose a uniform distribution of information in discourse (Uniform Information Density, (7–10). More informative units (in information-theoretic terms, i.e. larger surprisal in the current study) take more time in discourse, while redundant units can be uttered faster or eliminated (e.g. for optional words like complementizer *that* (7)).

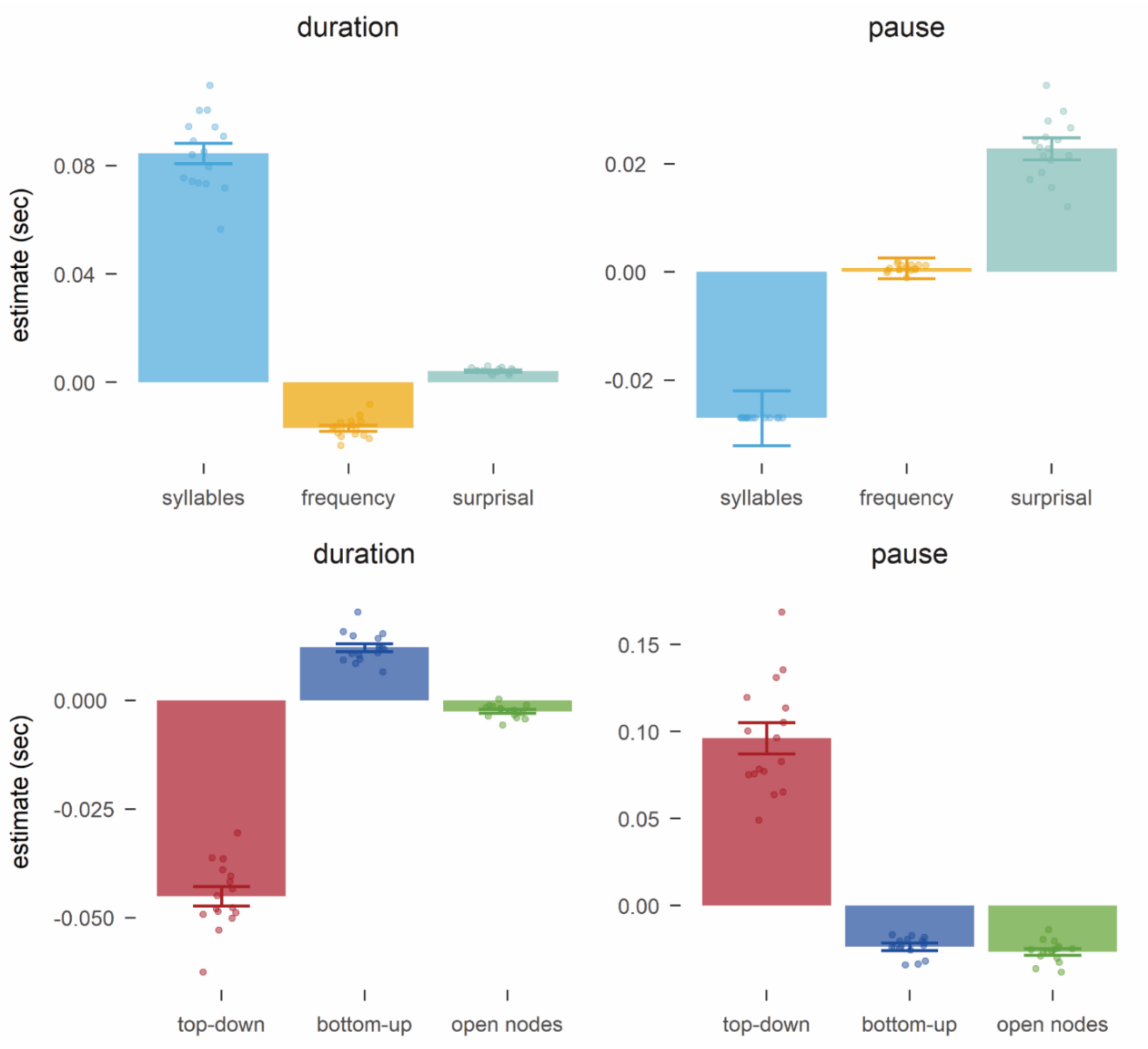

Fig. S4. Estimates in seconds of the effect of each predictor of word characteristics and phrase-structure building on word durations and pause length before word articulation. Error bars represent standard error of the mean. Individual points represent each participant's estimate as estimated by the random slopes. The model estimated identical random slopes for number of syllables on pause length for each participant.

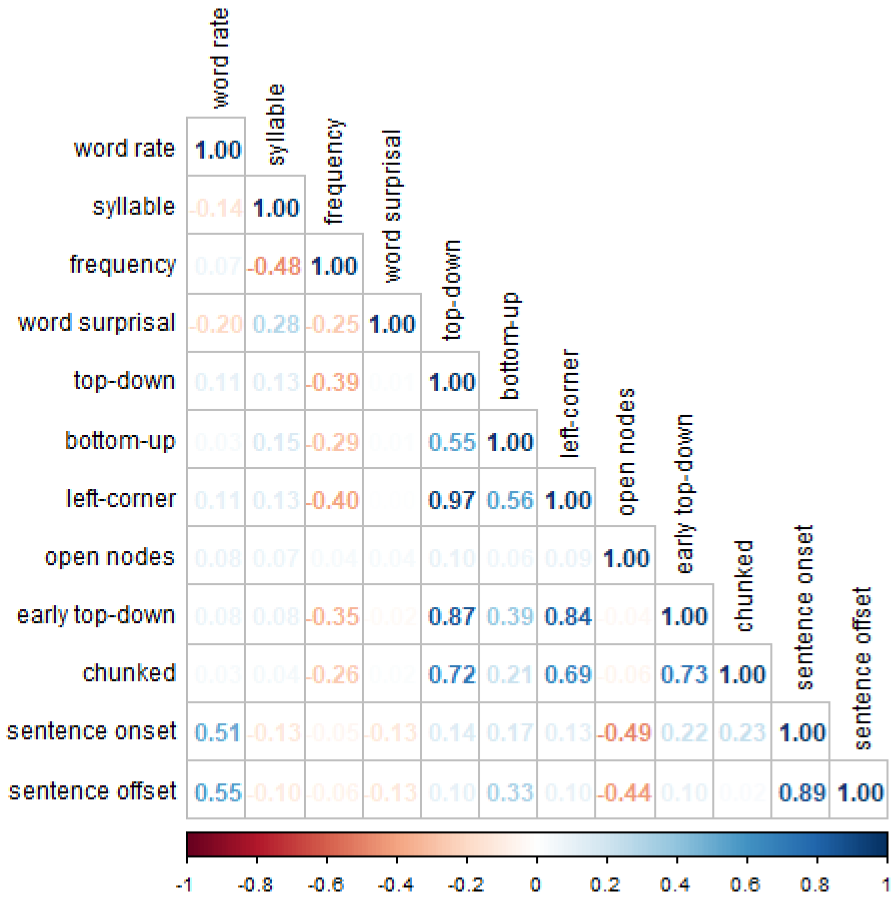

Fig. S5. Correlation matrix showing Pearson's r correlation among all predictors after they were convolved with the haemodynamic response function (corresponding to 16338 individual time points). Note that not all predictors were used in the same model.

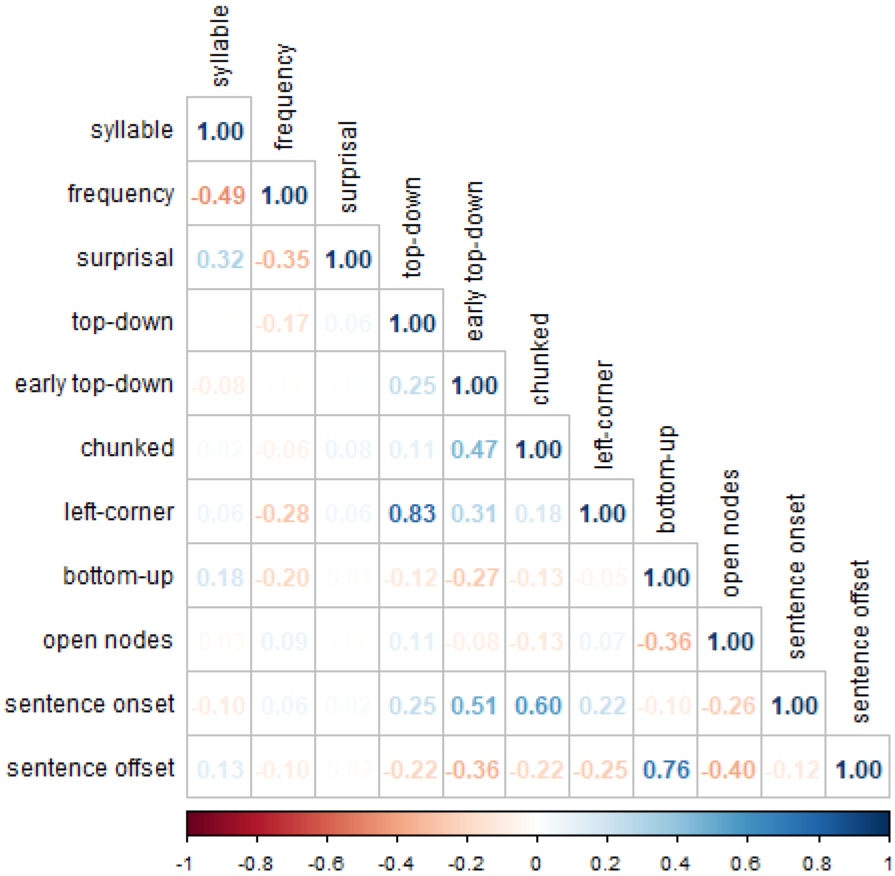

Fig. S6. Correlation matrix showing Pearson's r correlation among all predictors before they were convolved with the haemodynamic response function (51606 words across production and comprehension). Note that the word rate predictor is not present because it is a vector of 1s (after convolving it captures how often words are said/heard, based on their onset times).

fMRI data acquisition and preprocessing

Data Acquisition

The acquisition parameters were identical in the two datasets. MRI data was collected on a 3T full-body scanner (Siemens Skyra) with a 20-channel head coil. Functional images were acquired using a T2*-weighted echo planar imaging pulse sequence (TR 1500 ms, TE 28 ms, flip angle 64, whole-brain coverage 27 slices of 4 mm thickness, in-plane resolution 3 × 3 mm^2, FOV 192 × 192 mm^2). Anatomical images were acquired using a T1-weighted MPRAGE pulse sequence (0.89 mm^3 resolution).

fMRI preprocessing

Preprocessing was performed using *fMRIPrep* 20.2.6 (11), which is based on *Nipype* 1.7.0 (12, 13).

Anatomical data preprocessing

The T1-weighted (T1w) image was corrected for intensity non-uniformity (INU) with N4BiasFieldCorrection (14), distributed with ANTs 2.3.3 (15), and used as T1w-reference throughout the workflow. The T1w-reference was then skull-stripped with a *Nipype* implementation of the antsBrainExtraction.sh workflow (from ANTs), using OASIS30ANTs as target template. Brain tissue segmentation of cerebrospinal fluid (CSF), white-matter (WM) and gray-matter (GM) was performed on the brain-extracted T1w using fast (FSL 5.0.9 (16)). Brain surfaces were reconstructed using recon-all (FreeSurfer 6.0.1, (17)), and the brain mask estimated previously was refined with a custom variation of the method to reconcile ANTs-derived and FreeSurfer-derived segmentations of the cortical gray-matter of Mindboggle (18).

Functional data preprocessing

For each BOLD run, the following preprocessing was performed. First, a reference volume and its skull-stripped version were generated using a custom methodology of *fMRIPrep*. Susceptibility distortion correction (SDC) was omitted, because no fieldmap was acquired. The BOLD reference was then co-registered to the T1w reference using bbregister (FreeSurfer) which implements boundary-based registration (19). Co-registration was configured with six degrees of freedom. Head-motion parameters with respect to the BOLD reference (transformation matrices, and six corresponding rotation and translation parameters) are estimated before any spatiotemporal filtering using mcflirt (FSL 5.0.9, (20)). The BOLD time-series (including slice-timing correction when applied) were resampled onto their original, native space by applying the transforms to correct for head-motion. These resampled BOLD time-series will be referred to as *preprocessed BOLD in original space*, or just *preprocessed BOLD*. The BOLD time-series were resampled into standard space, generating a *preprocessed BOLD run in MNI152NLin2009cAsym space*. First, a reference volume and its skull-stripped version were generated using a custom methodology of *fMRIPrep*. Automatic removal of motion artifacts using independent component analysis (ICA-AROMA, (21)) was performed on the *preprocessed BOLD on MNI space* time-series after removal of non-steady state volumes and spatial smoothing with an isotropic, Gaussian kernel of 6mm FWHM (full-width half-maximum). The “aggressive” noise-regressors were collected and placed in the corresponding confounds file. Several confounding time-series were calculated based on the *preprocessed BOLD*: framewise displacement (FD), the derivative of the relative (frame-to-frame) bulk head motion variance (DVARS) and three region-wise global signals. FD was computed using two formulations following Power (absolute sum of relative motions, (22)) and Jenkinson (relative root mean square displacement between affines, (20)). FD and DVARS are calculated for each functional run, both using their implementations in *Nipype* (following the definitions by (22). Additionally, a set of physiological regressors were extracted to allow for component-based noise correction (*CompCor*, (23)). Principal components are estimated after high-pass filtering the *preprocessed BOLD* time-series (using a discrete cosine filter with 128s cut-off) for anatomical CompCor (aCompCor). For aCompCor, three probabilistic masks (cerebrospinal fluid (CSF), white matter (WM) and combined CSF+WM) are generated in anatomical space.

Table S1. Summary of model output of BOLD activity in production and comprehension. ROI1 refers to the contrast BA44 vs. BA45, ROI2 refers to the contrast BA44 & BA45 vs. pMTG. Mod stands for modality. AIC stands for Akaike Information Criterion, used for the production only models to determine model fit.

	BOLD				
Predictors	*Estimates*	*std. Error*	*CI*	*Statistic*	*p*
(Intercept)	-0.18	0.01	-0.20 – -0.15	-14.37	**<0.001**
word rate	0.52	0.05	0.42 – 0.61	10.84	**<0.001**
syllable	0.24	0.12	0.00 – 0.47	2.00	**0.046**
frequency	0.13	0.05	0.04 – 0.22	2.78	**0.005**
top-down	0.12	0.16	-0.19 – 0.43	0.75	0.452
ROI1	-0.01	0.01	-0.04 – 0.01	-1.04	0.298
ROI2	-0.00	0.01	-0.02 – 0.01	-0.36	0.719
mod1	-0.04	0.01	-0.06 – -0.02	-4.34	**<0.001**
bottom-up	-0.13	0.09	-0.31 – 0.05	-1.45	0.147
surprisal	0.16	0.02	0.11 – 0.21	6.62	**<0.001**
open nodes	0.03	0.02	-0.00 – 0.06	1.85	0.064
sent onset	-1.37	0.49	-2.32 – -0.41	-2.79	**0.005**
sent offset	1.42	0.55	0.35 – 2.50	2.59	**0.009**
top-down × ROI1	-0.02	0.10	-0.21 – 0.16	-0.25	0.801
top-down × ROI2	-0.20	0.06	-0.31 – -0.10	-3.68	**<0.001**
top-down × mod1	-0.45	0.16	-0.76 – -0.14	-2.87	**0.004**
ROI1 × mod1	-0.01	0.01	-0.04 – 0.01	-1.11	0.269
ROI2 × mod1	-0.01	0.01	-0.03 – 0.00	-1.82	0.069
ROI1 × bottom-up	-0.04	0.07	-0.18 – 0.10	-0.58	0.560
ROI2 × bottom-up	0.09	0.04	0.01 – 0.17	2.24	**0.025**
mod1 × bottom-up	0.29	0.09	0.12 – 0.46	3.33	**0.001**
mod1 × surprisal	0.01	0.02	-0.03 – 0.06	0.55	0.582
ROI1 × surprisal	0.08	0.02	0.04 – 0.12	3.80	**<0.001**
ROI2 × surprisal	0.01	0.01	-0.01 – 0.04	0.96	0.338

ROI1 × open nodes	0.02	0.01	0.00 – 0.04	2.14	**0.032**
ROI2 × open nodes	0.01	0.01	-0.00 – 0.02	1.50	0.134
mod1 × open nodes	0.05	0.02	0.02 – 0.08	3.00	**0.003**
ROI1 × sent onset	-1.01	0.59	-2.18 – 0.15	-1.71	0.088
ROI2 × sent onset	-0.38	0.34	-1.06 – 0.29	-1.12	0.263
mod1 × sent onset	-0.30	0.49	-1.26 – 0.66	-0.61	0.542
ROI1 × sent offset	1.39	0.61	0.20 – 2.57	2.29	**0.022**
ROI2 × sent offset	0.44	0.35	-0.24 – 1.13	1.26	0.208
mod1 × sent offset	1.27	0.50	0.29 – 2.25	2.54	**0.011**
top-down × ROI1 × mod1	-0.12	0.10	-0.31 – 0.06	-1.30	0.195
top-down × ROI2 × mod1	-0.12	0.06	-0.23 – -0.01	-2.22	**0.026**
(ROI1 × mod1) × bottom-up	0.05	0.07	-0.08 – 0.19	0.77	0.439
(ROI2 × mod1) × bottom-up	0.07	0.04	-0.01 – 0.15	1.66	0.097
(ROI1 × mod1) × surprisal	0.02	0.02	-0.02 – 0.06	0.81	0.417
(ROI2 × mod1) × surprisal	-0.02	0.01	-0.04 – 0.01	-1.35	0.178
(ROI1 × mod1) × open nodes	0.02	0.01	0.01 – 0.04	2.55	**0.011**
(ROI2 × mod1) × open nodes	0.01	0.01	0.00 – 0.02	2.35	**0.019**
(ROI1 × mod1) × sent onset	-1.10	0.59	-2.27 – 0.06	-1.86	0.063
(ROI2 × mod1) × sent onset	0.14	0.34	-0.53 – 0.81	0.41	0.679
(ROI1 × mod1) × sent offset	1.46	0.61	0.28 – 2.65	2.42	**0.016**
(ROI2 × mod1) × sent offset	0.19	0.35	-0.50 – 0.88	0.54	0.587
N_{subj}	52				
Observations	115743				
AIC	418480.764				

Table S2. Summary of model output of BOLD activity in production with top-down predictor. ROI1 refers to the contrast BA44 vs. BA45, ROI2 refers to the contrast BA44 & BA45 vs. pMTG. Mod stands for modality. AIC stands for Akaike Information Criterion, used for the production only models to determine model fit.

	BOLD				
Predictors	*Estimates*	*std. Error*	*CI*	*Statistic*	*p*
(Intercept)	-0.15	0.02	-0.18 – -0.11	-7.56	**<0.001**
word rate	0.46	0.05	0.35 – 0.56	8.33	**<0.001**
syllable	0.07	0.14	-0.21 – 0.35	0.50	0.620
frequency	0.08	0.07	-0.06 – 0.22	1.15	0.250
surprisal	0.15	0.03	0.09 – 0.22	4.78	**<0.001**
ROI1	0.00	0.01	-0.02 – 0.02	0.12	0.905
ROI2	0.00	0.01	-0.01 – 0.01	0.05	0.958
top-down	0.51	0.19	0.14 – 0.88	2.73	**0.006**
bottom-up	-0.42	0.10	-0.61 – -0.23	-4.41	**<0.001**
surprisal * ROI1	0.06	0.04	-0.01 – 0.14	1.75	0.080
surprisal * ROI2	0.03	0.02	-0.01 – 0.07	1.49	0.136
ROI1 * top-down	0.10	0.15	-0.20 – 0.40	0.67	0.501
ROI2 * top-down	-0.11	0.09	-0.28 – 0.07	-1.22	0.224
ROI1 * bottom-up	-0.10	0.10	-0.29 – 0.09	-1.03	0.303
ROI2 * bottom-up	0.03	0.06	-0.08 – 0.14	0.50	0.617
N_{subj}	16				
Observations	45099				
AIC	170821.3				

Table S3. Summary of model output of BOLD activity in production with early top-down predictor. ROI1 refers to the contrast BA44 vs. BA45, ROI2 refers to the contrast BA44 & BA45 vs. pMTG. Mod stands for modality. AIC stands for Akaike Information Criterion, used for the production only models to determine model fit.

	BOLD				
Predictors	*Estimates*	*std. Error*	*CI*	*Statistic*	*p*
(Intercept)	-0.15	0.02	-0.19 – -0.11	-7.88	**<0.001**
word rate	0.47	0.05	0.37 – 0.58	8.69	**<0.001**
syllable	0.07	0.14	-0.21 – 0.35	0.49	0.622
frequency	0.06	0.07	-0.08 – 0.21	0.90	0.370
surprisal	0.16	0.03	0.09 – 0.22	4.83	**<0.001**
ROI1	0.00	0.01	-0.02 – 0.02	0.12	0.906
ROI2	0.00	0.01	-0.01 – 0.01	0.06	0.956
early top-down	0.33	0.20	-0.06 – 0.72	1.64	0.100
bottom-up	-0.35	0.10	-0.55 – -0.16	-3.54	**<0.001**
surprisal * ROI1	0.06	0.04	-0.01 – 0.14	1.78	0.076
surprisal * ROI2	0.03	0.02	-0.01 – 0.07	1.42	0.155
ROI1 * early top-down	0.10	0.12	-0.14 – 0.35	0.85	0.397
ROI2 * early top-down	-0.17	0.07	-0.31 – -0.03	-2.34	**0.020**
ROI1 * bottom-up	-0.09	0.09	-0.27 – 0.08	-1.04	0.296
ROI2 * bottom-up	0.04	0.05	-0.07 – 0.14	0.68	0.494
N_{subj}	16				
Observations	45099				
AIC	170803.9				

Table S4. Summary of model output of BOLD activity in production with chunked top-down predictor. ROI1 refers to the contrast BA44 vs. BA45, ROI2 refers to the contrast BA44 & BA45 vs. pMTG. Mod stands for modality. AIC stands for Akaike Information Criterion, used for the production only models to determine model fit.

	BOLD				
Predictors	*Estimates*	*std. Error*	*CI*	*Statistic*	*p*
(Intercept)	-0.16	0.02	-0.19 – -0.12	-8.16	**<0.001**
word rate	0.49	0.05	0.38 – 0.59	9.01	**<0.001**
syllable	0.06	0.14	-0.22 – 0.34	0.40	0.691
frequency	0.05	0.07	-0.09 – 0.19	0.64	0.522
surprisal	0.15	0.03	0.09 – 0.21	4.63	**<0.001**
ROI1	0.00	0.01	-0.02 – 0.02	0.12	0.906
ROI2	0.00	0.01	-0.01 – 0.01	0.05	0.956
chunked top-down	0.18	0.11	-0.04 – 0.40	1.60	0.109
bottom-up	-0.28	0.08	-0.45 – -0.12	-3.38	**0.001**
surprisal * ROI1	0.06	0.04	-0.01 – 0.13	1.75	0.080
surprisal * ROI2	0.03	0.02	-0.01 – 0.07	1.50	0.133
ROI1 * chunked top-down	0.02	0.09	-0.15 – 0.19	0.21	0.830
ROI2 * chunked top-down	-0.05	0.05	-0.15 – 0.05	-0.98	0.326
ROI1 * bottom-up	-0.07	0.09	-0.24 – 0.10	-0.83	0.409
ROI2 * bottom-up	0.00	0.05	-0.10 – 0.10	0.04	0.971
N_{subj}	16				
Observations	45099				
AIC	170837.3				

Table S5. Summary of model output of BOLD activity in production and comprehension, including the temporal derivative (der) of all predictors of interest. ROI1 refers to the contrast BA44 vs. BA45, ROI2 refers to the contrast BA44 & BA45 vs. pMTG. Mod stands for modality. AIC stands for Akaike Information Criterion, used for the production only models to determine model fit.

	**BOLD**				
Predictors	*Estimates*	*std. Error*	*CI*	*Statistic*	*p*
(Intercept)	-0.17	0.01	-0.20 – -0.15	-13.83	**<0.001**
word	0.51	0.05	0.41 – 0.60	10.21	**<0.001**
syllable	0.24	0.12	0.01 – 0.46	2.02	**0.043**
frequency	0.13	0.05	0.04 – 0.22	2.72	**0.007**
top-down der	0.05	0.36	-0.64 – 0.75	0.15	0.879
ROI1	-0.01	0.01	-0.03 – 0.01	-0.95	0.343
ROI2	0.00	0.01	-0.01 – 0.02	0.25	0.804
mod1	-0.04	0.01	-0.06 – -0.02	-3.64	**<0.001**
bottom-up der	0.17	0.18	-0.19 – 0.53	0.94	0.346
surprisal der	0.07	0.12	-0.17 – 0.31	0.59	0.556
open nodes der	-0.03	0.06	-0.15 – 0.08	-0.60	0.547
sent onset der	-0.87	1.29	-3.40 – 1.66	-0.67	0.501
sent offset der	-1.56	1.19	-3.89 – 0.77	-1.32	0.188
top-down	0.08	0.17	-0.25 – 0.42	0.49	0.628
bottom-up	-0.10	0.12	-0.34 – 0.14	-0.83	0.409
surprisal	0.16	0.02	0.11 – 0.21	6.71	**<0.001**
open nodes	0.03	0.02	-0.01 – 0.06	1.66	0.097
sent onset	-1.33	0.56	-2.43 – -0.23	-2.38	**0.017**
sent offset	1.38	0.62	0.17 – 2.58	2.24	**0.025**
top-down der × ROI1	0.38	0.25	-0.12 – 0.87	1.49	0.135
top-down der × ROI2	-0.28	0.15	-0.56 – 0.01	-1.90	0.058
top-down der × mod1	-0.20	0.36	-0.90 – 0.50	-0.57	0.570
ROI1 × mod1	-0.01	0.01	-0.03 – 0.01	-0.98	0.325

ROI2 × mod1	-0.01	0.01	-0.03 – -0.00	-2.06	**0.040**
ROI1 × bottom-up der	0.05	0.17	-0.29 – 0.39	0.27	0.785
ROI2 × bottom-up der	0.06	0.10	-0.14 – 0.25	0.57	0.568
mod1 × bottom-up der	0.13	0.18	-0.23 – 0.48	0.69	0.491
mod1 × surprisal der	-0.42	0.12	-0.66 – -0.18	-3.40	**0.001**
ROI1 × surprisal der	-0.00	0.07	-0.13 – 0.12	-0.07	0.942
ROI2 × surprisal der	-0.20	0.04	-0.28 – -0.13	-5.44	**<0.001**
ROI1 × open nodes der	-0.00	0.05	-0.11 – 0.10	-0.05	0.963
ROI2 × open nodes der	0.09	0.03	0.03 – 0.15	2.81	**0.005**
mod1 × open nodes der	0.08	0.06	-0.03 – 0.20	1.42	0.155
ROI1 × sent onset der	-0.16	1.14	-2.39 – 2.07	-0.14	0.888
ROI2 × sent onset der	0.12	0.66	-1.17 – 1.41	0.18	0.855
mod1 × sent onset der	2.34	1.27	-0.15 – 4.84	1.84	0.066
ROI1 × sent offset der	0.87	1.24	-1.56 – 3.29	0.70	0.484
ROI2 × sent offset der	-2.07	0.71	-3.48 – -0.67	-2.90	**0.004**
mod1 × sent offset der	-2.29	1.17	-4.59 – 0.01	-1.95	0.051
ROI1 × top-down	-0.02	0.15	-0.31 – 0.26	-0.16	0.870
ROI2 × top-down	-0.40	0.09	-0.57 – -0.23	-4.71	**<0.001**
mod1 × top-down	-0.57	0.17	-0.90 – -0.23	-3.34	**0.001**
ROI1 × bottom-up	-0.04	0.13	-0.30 – 0.22	-0.29	0.769
ROI2 × bottom-up	0.29	0.08	0.14 – 0.44	3.82	**<0.001**
mod1 × bottom-up	0.40	0.12	0.16 – 0.64	3.28	**0.001**
mod1 × surprisal	0.02	0.02	-0.03 – 0.06	0.66	0.506
ROI1 × surprisal	0.08	0.02	0.04 – 0.12	3.79	**<0.001**
ROI2 × surprisal	0.01	0.01	-0.01 – 0.04	1.02	0.307
ROI1 × open nodes	0.02	0.01	-0.00 – 0.04	1.78	0.076
ROI2 × open nodes	0.01	0.01	-0.00 – 0.02	1.13	0.258
mod1 × open nodes	0.04	0.02	0.01 – 0.07	2.53	**0.011**

ROI1 × sent onset	-0.80	0.68	-2.12 – 0.53	-1.18	0.239
ROI2 × sent onset	-0.46	0.39	-1.22 – 0.31	-1.17	0.241
mod1 × sent onset	-0.18	0.56	-1.28 – 0.91	-0.33	0.744
ROI1 × sent offset	1.14	0.70	-0.23 – 2.51	1.63	0.103
ROI2 × sent offset	0.40	0.40	-0.39 – 1.19	0.98	0.326
mod1 × sent offset	0.98	0.58	-0.15 – 2.11	1.69	0.090
top-down der × ROI1 × mod1	-0.23	0.25	-0.73 – 0.26	-0.92	0.358
top-down der × ROI2 × mod1	0.58	0.15	0.29 – 0.87	3.97	**<0.001**
(ROI1 × mod1) × bottom-up der	-0.06	0.17	-0.40 – 0.28	-0.33	0.745
(ROI2 × mod1) × bottom-up der	-0.07	0.10	-0.26 – 0.13	-0.68	0.496
(ROI1 × mod1) × surprisal der	-0.25	0.07	-0.38 – -0.12	-3.81	**<0.001**
(ROI2 × mod1) × surprisal der	0.06	0.04	-0.02 – 0.13	1.56	0.119
(ROI1 × mod1) × open nodes der	-0.02	0.05	-0.13 – 0.08	-0.43	0.666
(ROI2 × mod1) × open nodes der	-0.10	0.03	-0.16 – -0.04	-3.24	**0.001**
(ROI1 × mod1) × sent onset der	-0.52	1.14	-2.76 – 1.71	-0.46	0.647
(ROI2 × mod1) × sent onset der	-1.22	0.66	-2.51 – 0.07	-1.85	0.064
(ROI1 × mod1) × sent offset der	-0.82	1.24	-3.24 – 1.61	-0.66	0.509
(ROI2 × mod1) × sent offset der	-0.78	0.71	-2.18 – 0.63	-1.08	0.278
(ROI1 × mod1) × top-down	-0.08	0.15	-0.37 – 0.20	-0.57	0.567
(ROI2 × mod1) × top-down	0.02	0.09	-0.15 – 0.18	0.21	0.836
(ROI1 × mod1) × bottom-up	0.02	0.13	-0.24 – 0.28	0.13	0.895
(ROI2 × mod1) × bottom-up	-0.09	0.08	-0.24 – 0.06	-1.16	0.246
(ROI1 × mod1) × surprisal	0.02	0.02	-0.02 – 0.06	0.83	0.408
(ROI2 × mod1) × surprisal	-0.01	0.01	-0.04 – 0.01	-0.91	0.363
(ROI1 × mod1) × open nodes	0.02	0.01	0.00 – 0.04	2.43	**0.015**
(ROI2 × mod1) × open nodes	0.02	0.01	0.01 – 0.03	3.22	**0.001**
(ROI1 × mod1) × sent onset	-1.41	0.68	-2.74 – -0.08	-2.08	**0.037**
(ROI2 × mod1) × sent onset	-0.45	0.39	-1.21 – 0.32	-1.15	0.249

(ROI1 × mod1) × sent offset	1.73	0.70	0.36 – 3.10	2.48	**0.013**
(ROI2 × mod1) × sent offset	0.83	0.40	0.04 – 1.62	2.06	**0.039**
N_{subj}	52				
Observations	115743				
AIC	418183.922				

Table S6. Summary of model output of the pause length preceding each word's production. AIC stands for Akaike Information Criterion, used for the production only models to determine model fit.

	pause length				
Predictors	*Estimates*	*std. Error*	*CI*	*Statistic*	*p*
(Intercept)	0.15	0.02	0.11 – 0.19	7.11	**<0.001**
frequency	0.00	0.00	-0.00 – 0.00	0.36	0.722
surprisal	0.02	0.00	0.02 – 0.03	11.22	**<0.001**
syllables	-0.03	0.01	-0.04 – -0.02	-5.36	**<0.001**
bottom-up	-0.02	0.00	-0.03 – -0.02	-10.36	**<0.001**
open nodes	-0.03	0.00	-0.03 – -0.02	-13.66	**<0.001**
top-down	0.10	0.01	0.08 – 0.11	10.66	**<0.001**
N_{subj}	16				
Observations	45079				
AIC	82830.780				

Table S7. Summary of model output of word duration. AIC stands for Akaike Information Criterion, used for the production only models to determine model fit.

	**word duration**				
Predictors	*Estimates*	*std. Error*	*CI*	*Statistic*	*p*
(Intercept)	0.27	0.01	0.25 – 0.29	28.22	**<0.001**
frequency	-0.02	0.00	-0.02 – -0.01	-15.44	**<0.001**
surprisal	0.00	0.00	0.00 – 0.01	9.38	**<0.001**
syllables	0.08	0.00	0.08 – 0.09	22.50	**<0.001**
bottom-up	0.01	0.00	0.01 – 0.01	12.61	**<0.001**
open nodes	-0.00	0.00	-0.00 – -0.00	-5.35	**<0.001**
top-down	-0.05	0.00	-0.05 – -0.04	-20.05	**<0.001**
N_{subj}	16				
Observations	45079				
AIC	-42830.283				

SI References

1. E. Fedorenko, P.-J. Hsieh, A. Nieto-Castañón, S. Whitfield-Gabrieli, N. Kanwisher, New Method for fMRI Investigations of Language: Defining ROIs Functionally in Individual Subjects. *Journal of Neurophysiology* **104**, 1177–1194 (2010).

2. J. R. Brennan, E. P. Stabler, S. E. Van Wagenen, W.-M. Luh, J. T. Hale, Abstract linguistic structure correlates with temporal activity during naturalistic comprehension. *Brain and Language* **157–158**, 81–94 (2016).

3. J. R. Brennan, C. Dyer, A. Kuncoro, J. T. Hale, Localizing syntactic predictions using recurrent neural network grammars. *Neuropsychologia* **146**, 107479 (2020).

4. C. Shain, I. A. Blank, M. van Schijndel, W. Schuler, E. Fedorenko, fMRI reveals language-specific predictive coding during naturalistic sentence comprehension. *Neuropsychologia* **138**, 107307 (2020).

5. R. M. Willems, S. L. Frank, A. D. Nijhof, P. Hagoort, A. van den Bosch, Prediction During Natural Language Comprehension. *Cerebral Cortex* **26**, 2506–2516 (2016).

6. M. Aylett, A. Turk, The Smooth Signal Redundancy Hypothesis: A Functional Explanation for Relationships between Redundancy, Prosodic Prominence, and Duration in Spontaneous Speech. *Lang Speech* **47**, 31–56 (2004).

7. T. F. Jaeger, Redundancy and reduction: Speakers manage syntactic information density. *Cognitive Psychology* **61**, 23–62 (2010).

8. T. F. Jaeger, R. Levy, Speakers optimize information density through syntactic reduction in *Advances in Neural Information Processing Systems*, (MIT Press, 2007).

9. H. Karimi, Greater entropy leads to more explicit referential forms during language production. *Cognition* **225**, 105093 (2022).

10. S. T. Piantadosi, H. Tily, E. Gibson, Word lengths are optimized for efficient communication. *Proceedings of the National Academy of Sciences* **108**, 3526–3529 (2011).

11. O. Esteban, *et al.*, fMRIPrep: a robust preprocessing pipeline for functional MRI. *Nature Methods*, 1 (2018).

12. K. Gorgolewski, *et al.*, Nipype: a flexible, lightweight and extensible neuroimaging data processing framework in Python. *Frontiers in Neuroinformatics* **5**, 13 (2011).

13. K. J. Gorgolewski, *et al.*, Nipype. *Software* (2018) https:/doi.org/10.5281/zenodo.596855.

14. N. J. Tustison, *et al.*, N4ITK: Improved N3 Bias Correction. *IEEE Transactions on Medical Imaging* **29**, 1310–1320 (2010).

15. B. B. Avants, C. L. Epstein, M. Grossman, J. C. Gee, Symmetric diffeomorphic image registration with cross-correlation: Evaluating automated labeling of elderly and neurodegenerative brain. *Medical Image Analysis* **12**, 26–41 (2008).

16. Y. Zhang, M. Brady, S. Smith, Segmentation of brain MR images through a hidden Markov random field model and the expectation-maximization algorithm. *IEEE Transactions on Medical Imaging* **20**, 45–57 (2001).

17. A. M. Dale, B. Fischl, M. I. Sereno, Cortical Surface-Based Analysis: I. Segmentation and Surface Reconstruction. *NeuroImage* **9**, 179–194 (1999).

18. A. Klein, *et al.*, Mindboggling morphometry of human brains. *PLOS Computational Biology* **13**, e1005350 (2017).

19. D. N. Greve, B. Fischl, Accurate and robust brain image alignment using boundary-based registration. *NeuroImage* **48**, 63–72 (2009).

20. M. Jenkinson, P. Bannister, M. Brady, S. Smith, Improved Optimization for the Robust and Accurate Linear Registration and Motion Correction of Brain Images. *NeuroImage* **17**, 825–841 (2002).

21. R. H. R. Pruim, *et al.*, ICA-AROMA: A robust ICA-based strategy for removing motion artifacts from fMRI data. *NeuroImage* **112**, 267–277 (2015).

22. J. D. Power, *et al.*, Methods to detect, characterize, and remove motion artifact in resting state fMRI. *NeuroImage* **84**, 320–341 (2014).

23. Y. Behzadi, K. Restom, J. Liau, T. T. Liu, A component based noise correction method (CompCor) for BOLD and perfusion based fMRI. *NeuroImage* **37**, 90–101 (2007).
